# The on-premise data sharing infrastructure e!DAL: Foster FAIR data for faster data acquisition

**DOI:** 10.1093/gigascience/giaa107

**Published:** 2020-10-22

**Authors:** Daniel Arend, Patrick König, Astrid Junker, Uwe Scholz, Matthias Lange

**Affiliations:** Leibniz Institute of Plant Genetics and Crop Plant Research (IPK) Gatersleben, Corrensstrasse 3, D-06466 Seeland, Germany; Leibniz Institute of Plant Genetics and Crop Plant Research (IPK) Gatersleben, Corrensstrasse 3, D-06466 Seeland, Germany; Leibniz Institute of Plant Genetics and Crop Plant Research (IPK) Gatersleben, Corrensstrasse 3, D-06466 Seeland, Germany; Leibniz Institute of Plant Genetics and Crop Plant Research (IPK) Gatersleben, Corrensstrasse 3, D-06466 Seeland, Germany; Leibniz Institute of Plant Genetics and Crop Plant Research (IPK) Gatersleben, Corrensstrasse 3, D-06466 Seeland, Germany

**Keywords:** research data management, FAIR principles, digital object identifier, plant genomics and phenomics

## Abstract

**Background:**

The FAIR data principle as a commitment to support long-term research data management is widely accepted in the scientific community. Although the ELIXIR Core Data Resources and other established infrastructures provide comprehensive and long-term stable services and platforms for FAIR data management, a large quantity of research data is still hidden or at risk of getting lost. Currently, high-throughput plant genomics and phenomics technologies are producing research data in abundance, the storage of which is not covered by established core databases. This concerns the data volume, e.g., time series of images or high-resolution hyper-spectral data; the quality of data formatting and annotation, e.g., with regard to structure and annotation specifications of core databases; uncovered data domains; or organizational constraints prohibiting primary data storage outside institional boundaries.

**Results:**

To share these potentially dark data in a FAIR way and master these challenges the ELIXIR Germany/de.NBI service Plant Genomic and Phenomics Research Data Repository (PGP) implements a “bring the infrastructure to the data” approach, which allows research data to be kept in place and wrapped in a FAIR-aware software infrastructure. This article presents new features of the e!DAL infrastructure software and the PGP repository as a best practice on how to easily set up FAIR-compliant and intuitive research data services. Furthermore, the integration of the ELIXIR Authentication and Authorization Infrastructure (AAI) and data discovery services are introduced as means to lower technical barriers and to increase the visibility of research data.

**Conclusion:**

The e!DAL software matured to a powerful and FAIR-compliant infrastructure, while keeping the focus on flexible setup and integration into existing infrastructures and into the daily research process.

## Introduction

The FAIR (Findability, Accessibility, Interoperability, and Reusability) principles, drafted by the FORCE11 workgroup in 2015 [1] and published in 2016 by Wilkinson et al. [2], are widely accepted and are increasingly adopted in management policies for research data. The scientific community is showing an increasing awareness of the scientific value of reusable research data. This has already resulted in the FAIR principles being formally accepted in several data management guidelines, e.g., in the Horizon2020 program [3] of the European Commission, and integrated into research funding policy [4,5]. Its technical implementation is supported by data repositories, which store and share research data in a FAIR manner. These can be classified into (i) general purpose data repositories, e.g., figshare [6], Zenodo [7], Dryad [8], and FAIRDOM [9]; (ii) core data deposition databases, e.g., ELIXIR deposition databases for life science data [10] and NCBI database resources [11]; and (iii) specific databases and repositories hosted by research institutes. All have in common that the research data have to be transferred by their owner from the place of data generation to these repositories. This involves considerable effort for data compilation, cleansing, homogenization, metadata enrichment, formatting, and upload. As a result, the published datasets are condensed and generally limited to insufficiently documented supplement material for publications in scientific journals. In the case that data should be submitted to database systems, e.g., the European Bioinformatics Institute (EBI) and NCBI core data resources, bioinformaticians are charged and trained to meet the specific submission requirements and support biologists. Examples are the preparation of data for submission to the EBI European Nucleotide Archive (ENA) [12,13], the European Variation Archive (EVA) [14], or the preparation of ISA-TAB–compatible data submission for plant phenotyping data [15,16]. Alternatively, institutes could set up project-related data repositories. This in turn requires skilled technicians and computer scientists, as well as long-term access to appropriate network and storage infrastructure. Such repositories frequently have a short lifetime, whether due to staff fluctuation, long-term maintenance costs, or resource consumption. Another reason may be that the repository’s niche is too specific to attract substantial data volume, which in turn strongly depends on policies and cost-benefit considerations.

**Figure 1:**
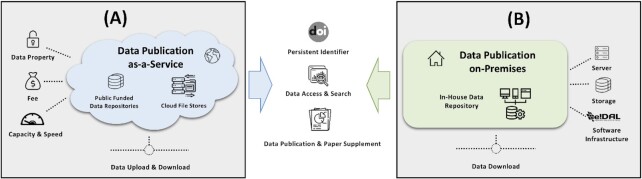
Data-publication-as-a-service vs data-publication-on-premises: both services feature FAIR data publication but differ in costs. The as-a-service model (A) costs a fee, involves delegation of data property control, and faces capacity limits in storage and data upload. The on-premises model (B) keeps data in-house but requires the availability of server and storage hardware as well as the installation of the e!DAL software.

**Figure 2:**
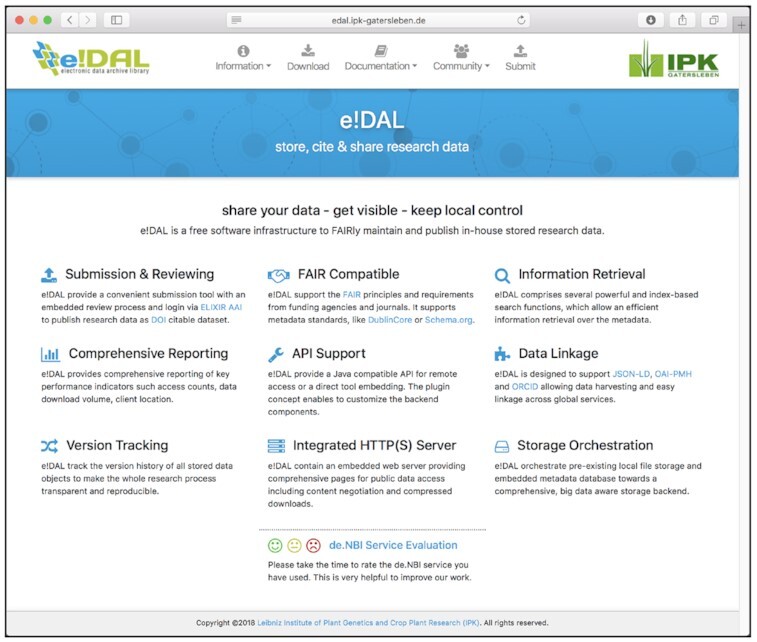
Screenshot of the e!DAL website, which provides a detailed description on how to use the e!DAL infrastructure software, as well as comprehensive examples for users and developers. Furthermore, some video tutorials and presentation recordings are available to lower the initial barriers to digging into the e!DAL infrastructure.

Thus, there is a need for an additional class of repositories that support data sharing for this class of research data by moving the infrastructure to the data. The concept is to apply an on-premises, infrastructure-to-the-data (I2D) principle. The basic idea of the I2D approach is shown in Fig. 1. In contrast to the conventional data publication pipelines to journal accepted databases, which usually involve a time-consuming data upload to an external platform and possibly additional costs depending on the required storage space, the underlying e!DAL software [17] uses a data publication layer to encapsulate an existing storage infrastructure. This layer is a broker to the DataCite [18] data publication service agent and provides an API and a tooling infrastructure for data submission, DOI delivery, reporting, and data quality review. This finally enables the assignment of DOIs with a minimal set of technical metadata, which are based on the DublinCore, to in-house stored data and their approved FAIR referencing by journals or data lookup services. The e!DAL website, which is shown in Fig. 2 provides, detailed information and examples about the installation and usage of the software.

As proof of concept, the Plant Genomics and Phenomics Data Repository (PGP) was implemented [19] using the e!DAL infrastructure software to publish digital plant genetic resources (PGR) [20] according to the FAIR principles. PGRs are the basis of food security and consist of diversity of seeds and planting materials of modern cultivars and crop wild relatives [21]. Approximately 7 million PGR accessions are conserved in genebank collections worldwide. The valorization of PGRs through genotyping and phenotyping is of special focus in the public and private sectors [22,23]. The data management of digital PGRs is identified as one of the most important challenges for a long-term strategy to enhance the productivity, sustainability, and resilience of crop varieties and agricultural systems. In contrast to successful studies on genomics-assisted genebank management and the use of germplasm collections [22], the special focus of the PGP repository is the publication of buckets of research data that do not fit into general purpose sharing platform or core data deposition databases owing to their volume, objective, structure, or incomplete analysis. Examples are primary data from imaging, field phenotyping, single-nucleotide polymorphism matrices, 3D plant models, metabolite screenings, and environmental sensor data. The experience gained during the 4-year operation of the repository has led to a growing acceptance of this approach for the publication of digital PGRs collected in the context of the German Federal *ex situ* Genebank of Agricultural and Horticultural Crop Species [24]. This experience and the addition to the *list of service* in the European life-sciences Infrastructure for biological Information ELIXIR [25] resulted in novel features, which were implemented with the aim of further improving its acceptance and enabling increased sharing of digital PGRs. After an update to the state of the art, the new features of the e!DAL data sharing software and its application for the publication of digital PGRs are explained.

## Related Work

Just as we have many different data types from several domains, there are also a variety of domain-specific archives and information systems. Most of them evolved over many years, and they are widely accepted by the research community [26]: ENA for genomic data [27], UniProt for protein data [28], PRIDE for proteome data [29], BioModels for systems biology data [30], and many more. As a guideline, research journals and other publishers require the sustainable publication of data according to FAIR criteria. For this purpose, established domain-specific databases or the use of long-term committed data repositories is recommended. In order to avoid data getting lost in the diversity of archives, there are several registries such as re3data.org or FAIRsharing.org, as well as consortia such as GFBio, which collect and categorize repositories to help researchers find suitable storage for their data.

Infrastructure programs such as the European Open Science Cloud (EOSC) [4] and the European life-sciences Infrastructure for biological Information (ELIXIR) [31] coordinate maintenance and interoperability of research data repositories as federated services by member organizations and hosting institutions. Furthermore, the ELIXIR organization has the aim of establishing a stable and sustainable infrastructure for biological information. In doing so, they define important core resources and deposition databases as a support of the research community [10] such as BRENDA [32] or SILVA [33]. Most of these systems accept only very specific datasets and require specialized metadata based on schemes that have been improved by the community over years. Unfortunately, there are several, mostly relatively new data types, e.g., plant phenotypic data, which currently do not fit into existing databases, mainly because of their strong heterogeneity and high volume. Public data sharing services such as figshare or DRYAD provide an alternative solution for publishing these datasets. They are easy to use and have a comprehensive functionality, including such features as supporting version control and the assignment of persistent identifiers. One important deficiency of such services is the limited free space, which is usually enough for sharing some reduced graphics or aggregated tables but not for storing large datasets. Furthermore, the establishment and configuration of an own in-house infrastructure based on existing software packages such as CKAN or Dataverse could overcome this shortcoming, but they require quite a lot of technical prerequisites and know-how.

## Infrastructure

To lower the technical barriers and minimize the effort for scientists to archive and share their research data, we developed the generic e!DAL software infrastructure [17]. The usual “data-publication-as-a-service” procedure includes the transfer of selected datasets to external databases and storage infrastructures after data generation and analysis. In this way research data can be referenced in a future research publication, as shown on the left side of Fig. 1A. In contrast the e!DAL infrastructure provides a “data-publication-on-premises” approach, which enables the publication of locally stored high-volume research data through the assignment of widely accepted and long-term stable DOIs. This is illustrated on the right side of Fig. 1B. Using DOIs for referencing provides multiple advantages for sharing and accessing research data. Besides adding them as supplements to a research article they can also be the basis for a comprehensive data paper [34]. Furthermore, the well-connected infrastructure of the DataCite consortia strongly increase the visibility of the research data assigned with a DOI. It is automatically linked with the ORCID account of the authors and can be found via DataCite Search and other common search engines or can be harvested via the Open Archives Initiative Protocol for Metadata Harvesting (OAI-PMH) interface.

**Figure 3:**
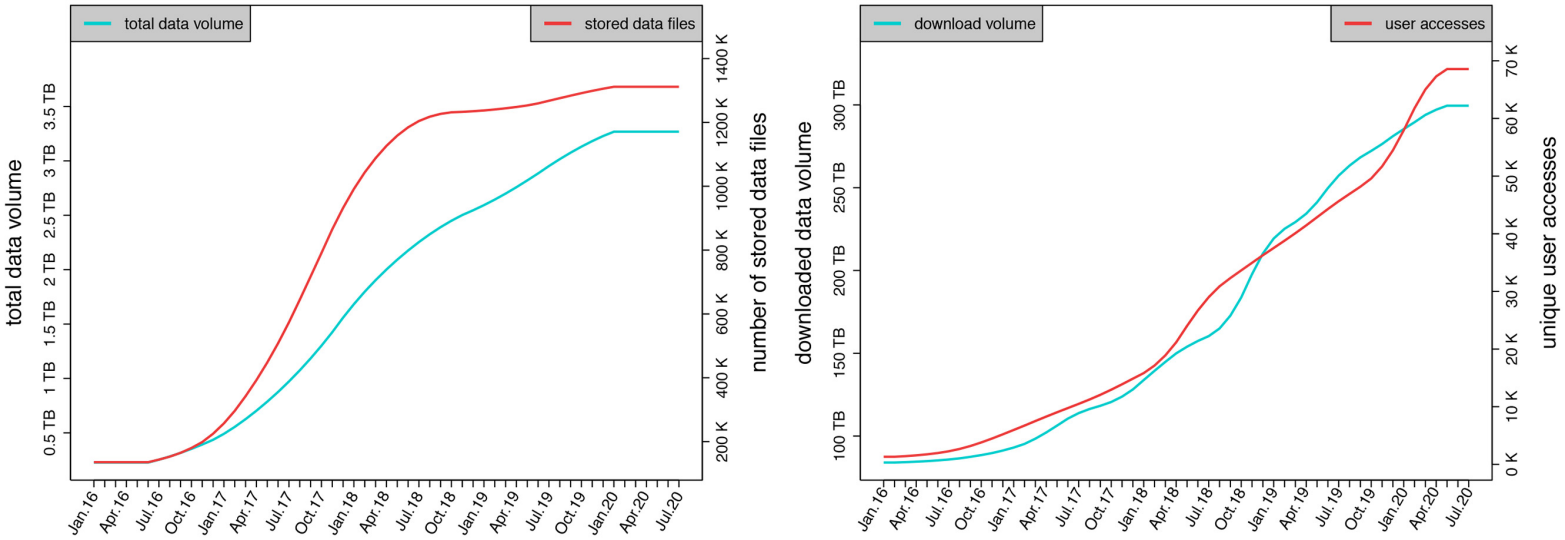
Access numbers and stock overview of the eDAL-PGP repository since 2016. *Left:* The development of the data volume and number of stored files, which were provided by the repository. *Right:*The constantly increasing number of accesses and downloads of published datasets.

Based on the e!DAL infrastructure, the Leibniz Institute of Plant Genetics and Crop Plant Research (IPK) Gatersleben and the German Plant Phenotyping Network (DPPN) jointly have initiated the PGP [19] as a powerful infrastructure for the publication of comprehensive plant genomics and phenomics research data. The repository covers in particular cross-domain datasets, which are not being published in public repositories for reasons of data volume or data domain, such as phenotyping images, genotyping data, visualizations of morphological models, and data from mass spectrometry, as well as software and related documents. In doing so, PGP currently provides 200 data records, which can be referenced via DOIs and are annotated with technical metadata. These records comprise >1.4 million files with an overall volume of >2.6 TB (see Fig. 3). To ensure data discoverability, PGP provides landing pages with JSON-LD formatted metadata and is therefore discoverable through data web crawler services that follow the Schema.org recommendations, such as those of Google, Microsoft, and Yandex. Furthermore, e!DAL implements the OAI-PMH. To support scientists in disseminating their research data the PGP infrastructure is accepted as institutional repository for the journals *Scientific Data* (Nature Publishing Group) and *GigaScience* (Oxford Academic) and is registered in re3data.org, FAIRsharing.org, OpenAIRE, and DataCite.

The benefits of this wide support of data discovery enabling technologies and data publication in general is proven by the steadily increasing number of dataset accesses. By June 2020, PGP delivered 300 TB of data and the provided datasets have been accessed by 100,000 unique clients.

## Improvements

The following section sums up the main enhancements and updates of the e!DAL infrastructure, which comprise new general features, comprehensive changes of several front-end components, and important performance improvements. Furthermore, an extensive update due to the latest changes in the Java programming languages and an improved build and deployment process is described.

### Performance

After releasing the first productive version of the PGP repository in 2015, we received many diverse data submissions from several research domains and with very heterogeneous data files. Since then we have recognized that the e!DAL infrastructure software scales well and is able to handle millions of data files, which confirms previous calculations and performance tests [17]. However, it became apparent that sometimes the performance decreases, e.g., for uploading comprehensive datasets with several hundred thousands of small files. Because this is a very common case, e.g., for plant phenotyping datasets, it was necessary to improve the implementation of the e!DAL infrastructure. Some major performance improvements are described subsequently.

One important feature of e!DAL is the automatic calculation of several essential technical metadata, such as the MIME-Type, the data volume, and the checksum of every file when storing new datasets. This is convenient because the user does not need to provide this information on his or her own, but of course these computations are a resource- and runtime-intensive process. Therefore, the functionality to determine the aforementioned technical metadata and the procedure to transfer the actual binary data have been improved to achieve parallel processing of multiple files. This results in better performance especially on today’s multi-core systems. Furthermore, we optimized several settings for the streaming buffer size and the remote transfer to improve the memory usage and the upload performance for the case of numerous small files. In addition, the checksum calculation was updated to use the more collision-resistant SHA-256 algorithm instead of the older and unsecure MD5 function.

### New features

The previous version of the e!DAL infrastructure already fulfilled several recommendations of the FAIR data principles, such as the support of standardized metadata based on the DublinCore schema and the provision of persistent DOIs for accessing and referencing of research datasets. The e!DAL infrastructure has been further updated to optimize its usability and the general user experience. Additional features were implemented to increase the visibility of published data and the acceptance of the infrastructure, which ultimately made it even more FAIR compliant. Thereby the road map for scholarly data repositories [35] was taken into account. Hereinafter, the most important extensions are described.

#### ORCID

To enable specific research data files to be efficiently found and accesssed across millions of datasets, persistent identifiers such as DOIs or URNs are very helpful and well established. Nevertheless, the research community is quite large, and sometimes it is difficult to distinguish data authors because of similar names or to identify the same researcher after a change of affiliation. With the Open Researcher and Contributor ID (ORCID) there is an easy and persistent solution to uniquely identify authors and to solve issues with name ambiguity [36]. An important advantage is its interdisciplinarity because ORCID is used across nearly all research domains and organizations; e.g., by mid-2019 in Germany there were already 150,000 ORCIDs registered [37]. Linking authors with publications, affiliations, or funding agencies helps to find relationships between researchers and their work and the corresponding research data. Because the e!DAL infrastructure is generic and suitable for different kinds of research data, the ORCID system gives us an ideal solution to identify authors and improve the collected metadata for published datasets. Furthermore, the authors and their research data will gain better visibility due to the connection between the ORCID infrastructure and infrastructure of the DataCite consortium that is handling the DOIs.

To add the possibility for assigning an ORCID to every data creator or contributor in the e!DAL infrastructure, the original PERSON data type [17] in the e!DAL metadata schema was extended. e!DAL uses the REST API of the ORCID registry to provide the possibility to search for the ORCID of a given name. In addition, it can be validated whether an entered ORCID belongs to the corresponding name to prevent accidentally linking with a wrong ORCID. All these API functions were integrated into the graphical user interface of the data submission tool for the PGP repository. Furthermore, the content pages of published and DOI linked datasets were improved to provide direct links to the ORCID profiles of the associated authors and contributors of the data.

#### JSON-LD and DC metatags

Another method of making research data interoperable as well as machine readable is to embed the describing metadata using JavaScript Object Notation for Linked Data (JSON-LD) format. This approach provides comprehensive possibilities to harvest and reuse research data. JSON-LD is a data serialization and exchange method and was developed to be easily embeddable into various systems for providing interoperable web services [38]. The dynamic HTML templates for the content pages of the embedded webserver of e!DAL, which provides the URLs for resolving the assigned DOIs, have been extended accordingly.

<script type ="application/ld+json">

{

"@context":"http://schema.org",

"@type":"Dataset",

"@id":"https://doi.org/10.5447/IPK/2016/7",

"name":"Raw images files from quantitative  monitoring of...",

"publisher":{

"@type":"Organization",

"name":"IPK Gatersleben"

},

"description":"This dataset contains 30426 raw   image files...",

"keywords":"high throughput plant phenotyping, growth protocol...",

"inLanguage":"en",

"author":[

{

"@type":"Person",

"givenName":"Astrid",

"familyName":"Junker",

"address":"IPK Gatersleben"

}

],

"contributor":[

{

"@type":"Person",

"givenName":"Thomas",

"familyName":"Altmann",

"address":"IPK Gatersleben"

}

]

}

**Listing 1**. Reduced example of the JSON-LD data from the content page of a DOI assigned with e!DAL, which is stored in the PGP repository.

Listing 1 shows an example for the JSON-LD description of a dataset in the PGP repository. The attributes are based on the Schema.org ontology, which is a well-established and community-driven vocabulary used to structure digital data on websites. It is used and harvested by several common search engines [39] and provides interoperability between datasets from separated resources and platforms.

Another alternative to JSON-LD is so-called HTML metatags. They are embedded in the <head> section of an HTML document and also allow harvesting of metadata and the description of connections between datasets from different infrastructures. As the metadata schema of the e!DAL infrastructure is already inspired by the DublinCore metadata schema [40], the embedded HTML templates for the content pages of published datasets were extended to also provide the technical metadata of every object as HTML metatags (see Listing 2).

<meta name="DC.Title" content="Screening of wild potato genetic...">

<meta name="DC.Identifier" content="https://doi.org/10.5447/IPK/2019/1">

<meta name="DC.Publisher" content="e!DAL - Plant Genomics and Phenomics...">

<meta name="DC.Language" content="en">

<meta name="DC.Description" content="This data set contains results of...">

<meta name="DC.Rights" content="CC BY-NC-SA 4.0">

<meta name="DC.Creator" content="Bachmann-Pfabe, Silvia...">

<meta name="DC.Contributor" content="Dehmer, Klaus...">

<meta name="DC.Subject" content="Phytophthora infestans">

<meta name="DC.Subject" content="germplasm collection">

**Listing 2**. Reduced example of the DublinCore Meta-Tags from the content page of a DOI assigned with e!DAL.

#### Content negotiation

**Figure 4:**
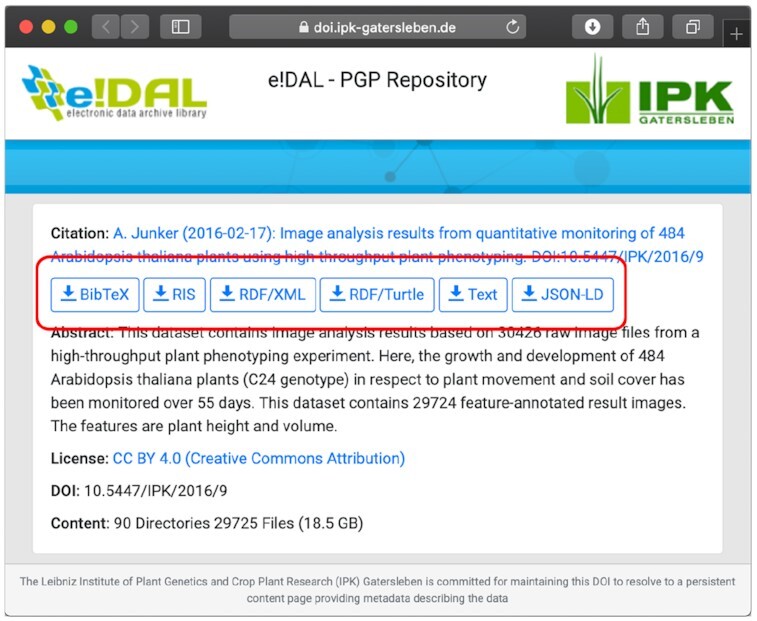
Screenshot of example datasets in the PGP repository. The red rectangle outlines the e!DAL embedded functions for content negotiation.

Persistent DOIs provide a solution for long-term stable resolvability and referencing of all published datasets. In addition, for several reasons such as citing the datasets or harvesting the metadata, it is necessary to provide content negotiation to serve resources in different formats. Therefore the possibility of getting different representations of the public datasets stored in an e!DAL infrastructure was implemented and can be used by several export functions, which were added on the corresponding content pages as shown in Fig. 4. They provide the option to get textual representations, citation formats such as BibTex or RIS, and linked data formats such as Schema.org/JSON-LD and RDF for every dataset. Because the DataCite service already provides a content negotiation feature, it was not necessary to implement a separate function for the embedded webserver of e!DAL. Instead, the HTTP handler uses the provided function for the different formats via a REST call and redirects the responses to the e!DAL infrastructure.

#### Elixir AAI

The e!DAL infrastructure provides a flexible and embedded security concept based on the Java Authentication and Authorization Service (JAAS). To provide research data management and publication capabilities to a wide range of users from universities, research institutes, or other organizations, a new login module using the ELIXIR Authentication and Authorization Infrastructure (AAI) [41] was implemented. It was designed to provide a single sign-on service for authenticating researchers to services that are a part of the ELIXIR portfolio. Doing so, it combines the huge amount of existing organizational identity providers from institutes that are associated with ELIXIR under 1 roof.

**Figure 5:**
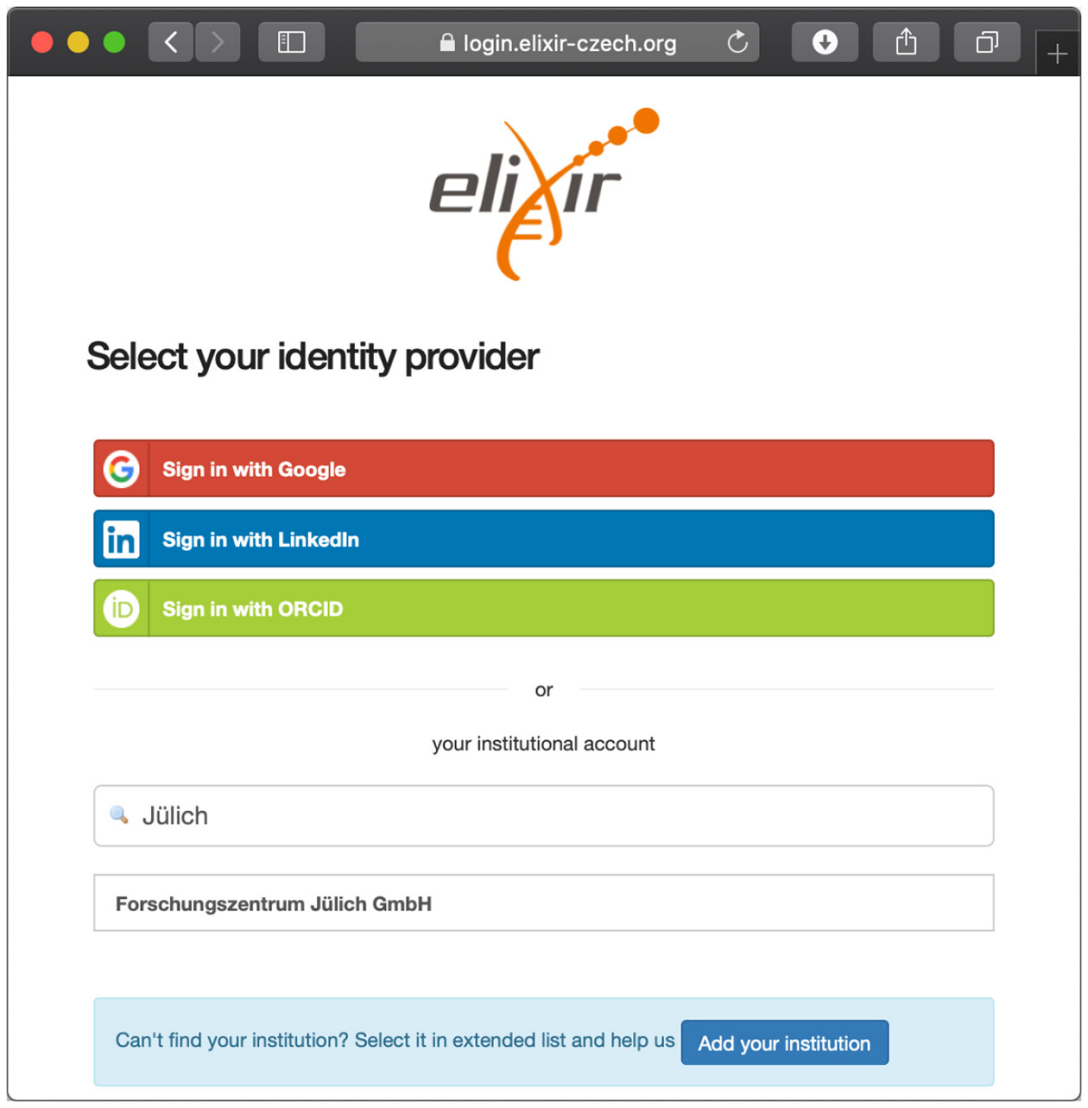
Integration of the ELIXIR AAI login dialog into the e!DAL infrastructure. In addition, the ELIXIR AAI provides a large collection of institutional identity providers, which can be easily found via the small search box, as well as several third-party login opportunities based on Google, ORCID, or LinkedIn. This provides the users a flexible authentication infrastructure.

The new e!DAL login module follows the OAuth protocol [42] to authenticate users over the ELIXIR AAI and automatically receive their e-mail address, which is necessary for communication between the data-submitting researcher and the reviewers involved in the embedded review process. Furthermore the e-mail address is used as a kind of internal ID to authenticate the user within the e!DAL security system [17]. As the first use case, the new ELIXIR AAI–based login was integrated into the PGP repository to open the infrastructure and the data submission process for offering the service to a wide range of researchers without the need of creating a separate account. The ELIXIR AAI allows researchers to use their existing organizational accounts (see Fig. 5), which lowers the barrier to use the infrastructure and to reach a larger group of data providers.

Furthermore, with the opportunity to use the ELIXIR AAI, the already low effort necessary to establish additional e!DAL installations was reduced. Therefore at the end of 2018 an additional e!DAL-based repository at the Jülich Plant Phenotyping Center (JPPC) was established using the ELIXIR AAI login provider.

#### Amended front end

The Apache Velocity template engine is used to render all HTML-based content of the e!DAL embedded webserver such as the landing pages of published datasets and e-mail messages. This prevents the infrastructure from storing a massive amount of very similar websites and text drafts, which saves storage and provides high performance for delivering content via the HTTP handler. All websites are provided dynamically on demand and created from only a few reusable templates.

**Figure 6:**
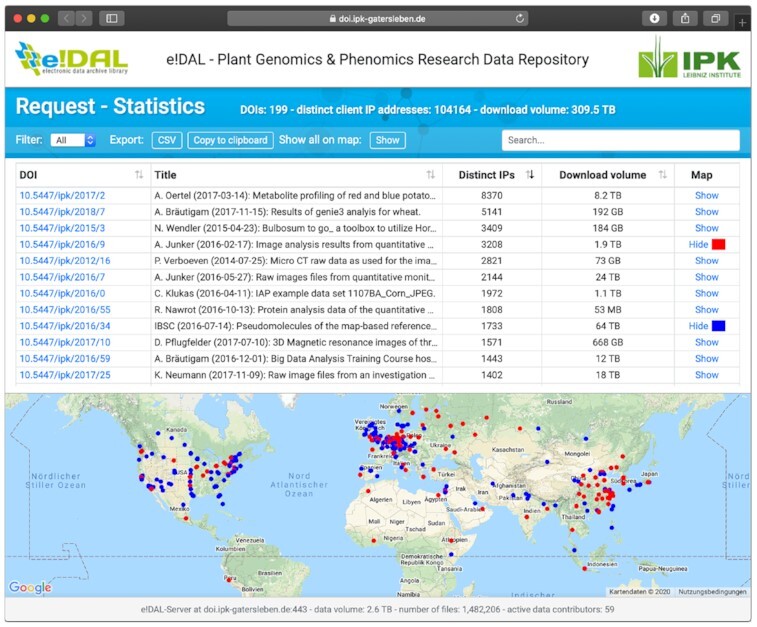
Example screenshot of the e!DAL embedded report page for the e!DAL-PGP repository showing the new layout and user interface. Several simple filter and search possibilities make it easier to look for specific datasets. An integrated world map gives the user an idea about the dissemination of the data.

For the latest e!DAL version all content pages and the underlying templates were fully redesigned to provide a pleasing visual look and functional user experience. Using front-end frameworks and libraries like BootStrap and jQuery ensures that the user interface is responsive and works on both modern desktop browsers as well as mobile devices. Fig. 6 shows the new layout as an example screenshot of the embedded access statistic page of the PGP repository. Together with the new design for the front-end components of e!DAL, the project website was also renewed to provide comprehensive information for the user and for developers in a more concise manner.

### Deployment and usability

Since the last major release of the e!DAL infrastructure software many optimizations and several new functionalities, which are described in the previous sections, have been implemented. Together with these improvements, changes in the general build and release process and in the usability have also been integrated. The most relevant of them are explained subsequently.

#### Gradle multi-build project

After using the Maven build system Build System for several years for developing and releasing the e!DAL software components a change to the Gradle build tool was made. Owing to the constantly increasing size of the project and the source code—because of new functionalities, several extensions, and additional unit tests to guarantee a high software quality—the build process using Maven takes quite a long time. This makes the regular release of stable versions very time-intensive. Furthermore, the build configuration became more complex and difficult to maintain. Gradle is strongly focused on a fast and specific build cycle. It supports multi-core systems to a high degree and allows, e.g., the execution of several test suites in parallel. With the change to the build infrastructure, we also decided to redesign the entire project build hierarchy and created a multi-build project for the e!DAL infrastructure. It contains the main API components including the reference implementation, as well as the components for the server-client architecture, which is directly based on this core implementation. This approach massively accelerates the build time, simplifies the maintenance, and allows a more frequent deployment of new versions. The project is now available in a new BitBucket repository.

Nevertheless, the API is still released as an artifact in the central Maven Repository and can be integrated into other software projects using Maven or Gradle, as shown in Listing 3.

-------- Maven - 'pom.xml' --------

<project>

<dependencies>

<dependency>

<groupId>de.ipk-gatersleben</groupId>

<artifactId>eDAL-MetaDataAPI|eDAL-MetaDataAPI-  Server | eDAL-MetaDataAPI-Client</artifactId>

<version>3.0.2</version>

</dependency>

<dependencies>

</project>

------- Gradle - 'build.gradle' -----

repositories {

mavenCentral()

}

dependencies {

compile 'de.ipk-gatersleben:eDAL-MetaDataAPI: 3.0.2'

compile 'de.ipk-gatersleben:eDAL-MetaDataAPI- Server:3.0.2'

compile 'de.ipk-gatersleben:eDAL-MetaDataAPI- Client:3.0.2'

}

**Listing 3**. Integration of the e!DAL components into the configuration of an Apache Maven or Gradle based project.

#### Operating system–specific executables

Owing to the complete new development and release cycle by Oracle, the Java programming environment, which is the basis for the e!DAL infrastructure, has changed a lot in recent years. In addition, the comprehensive redesign and reconstruction of the language itself, including the introduction of the new module concept and the removal of popular and formerly native APIs and frameworks such as JavaFX and the Java Network Launching Protocol (JNLP), which was the basis for Java web start applications, were some very substantial changes. This strongly influences the e!DAL implementation because they were also a significant part of the previous version. Unfortunately this impeded the further development of e!DAL infrastructure at some points because a lot of the frameworks and libraries needed several months to update their code to be compatible with the latest Java versions. With the new version 3.0.0 the e!DAL infrastructure is fully based on the Java Runtime Environment (JRE) 12. Therefore some comprehensive changes were necessary. To run e!DAL with the different existing runtimes, e.g., the official runtime from Oracle, but also the alternative and widely used OpenJDK, it was necessary to integrate the JavaFX library directly into the implementation. This increases the actual size of the API package, but it makes the infrastructure much more compatible and even more independent from the system preconditions than before.

The removal of the support for the popular and well-known JNLP was also a considerable challenge because the Java webstart tool was used to give the user an intuitive and platform-independent way to run the graphical data submission tool. Nevertheless this solution also involves some shortcomings like the need to provide an installed and compatible Java runtime. With the recently developed jpackage Java provides a powerful tool to pack self-contained applications along with a suitable JRE. We used jpackage to create a full image of the e!DAL data submission tool together with a reduced JRE, which contains only the necessary Java modules and provides separate executables for the most common operating systems (Windows, Unix, MacOS). This provides convenient usability for the data submitter and makes the infrastructure yetagain more compatible and independent from the given system preconditions of the users.

#### Web-based submission application

**Figure 7:**
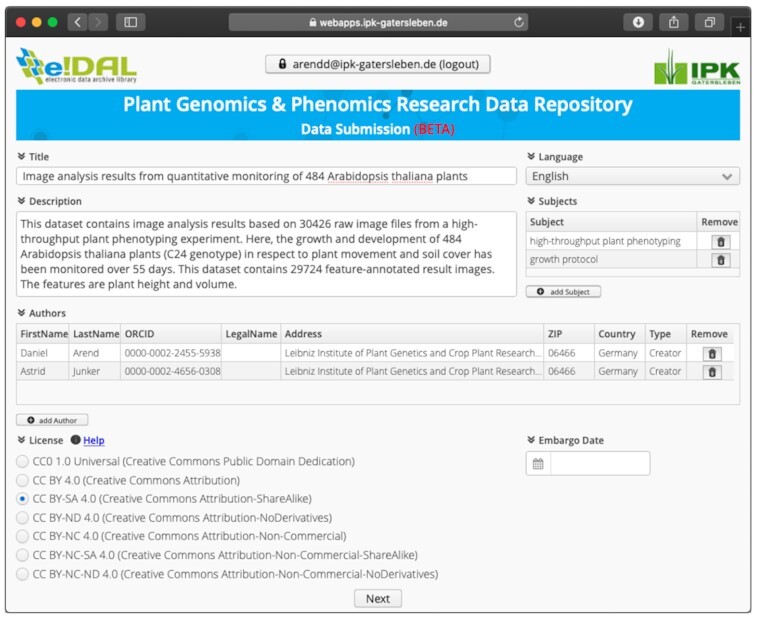
Example screenshot of the web-based submission application for the e!DAL-PGP repository. The form-like user interface is very intuitive and similar to the desktop application. It helps the user to fill in the needed metadata by executing several checks to guarantee completeness.

In parallel to the update process due to the previously mentioned changes in the JRE and the development of the build process to create the self-executable applications for the submission dialog, a new web-based application was implemented to provide an alternative opportunity to upload research data to an e!DAL-based infrastructure. The goal was the deployment of a user-friendly web application with functionality similar to that of the corresponding desktop tool but without the need to download the application as an executable or additional plugins. The Vaadin framework for Rich Internet Applications (RIA) was used for the implementation. Fig. 7 shows a screenshot of the web application. By using several REST APIs, e.g., from the ORCID Registry or the ELIXIR AAI, a lightweight application could be created providing the same functionality as the full desktop client. Furthermore, users now also have the option to submit research data from mobile devices or other browser-compatible devices. The only small shortcoming of the data submission via the web application is that currently not all browsers support the upload of comprehensive file folders. The latter is only possible if a recent version of Google Chrome or Mozilla Firefox is used. Other web browsers only allow the upload of single files.

## Results

In this article the basic overall “on-premises" data management and publication concept of the e!DAL infrastructure, as well as several new features and technical developments, are presented. As a result, e!DAL has matured into a comprehensive and FAIR-compliant infrastructure, while always keeping the focus on simple and flexible set-up and integration into existing infrastructures and into the daily research process. With the described “bring the infrastructure to the data" approach, it differs fundamentally from generic publication platforms like figshare or DRYAD, which can produce, depending on the needed storage, considerable financial costs and time costs for transferring the data. e!DAL allows the use of available in-house storage capacity, without the need of complex requirements and technical infrastructure or comprehensive adaptations. All functionalities are already included and the provided reference implementation contains required components, such as a database or a web server. This is a crucial advantage in comparison with other similar software infrastructures, such as DataVerse or CKAN, and lowers the barrier to establish a publication infrastructure even for small research institutions with limited resources and expertise. Thereby the FAIR criteria can be fulfilled by several e!DAL functions and components:

- **Findable:**By providing embedded and machine-readable metadata based on standardized established formats, the e!DAL published datasets can be easily found using common search engines such as Google or the DataCite Metadata Search. Owing to the well-established use of DOIs, the DataCite consortium is also involved in several projects and interacts with such systems as ORCID, CrossRef, and Scholix. This further improves the findability of e!DAL datasets.
- **Accessible:**e!DAL fully supports the use of DOIs as persistent identifiers to guarantee long-term stable availability of published datasets. The DataCite resolver for the DOIs allows simple access to the data and reference datasets, e.g., in a research article or as part of data publication. If the storage location of the underlying data changes, the corresponding DOI remains stable and allows uninterrupted access to the data by updating the resource path. At that point e!DAL's embedded web server takes care that every published DOI is accessible via a comprehensive content page. It provides the opportunity to navigate through the dataset and download certain files, as well as access to the metadata and a direct link to the ORCID registry.
- **Interoperable:**To provide interoperable datasets and to allow the aggregation of information about the relationship of datasets from different sources, the e!DAL infrastructure supplies embedded metadata on the content pages of every data object. They are stored using standardized formats and vocabularies such as JSON/LD or Schema.org.
- **Reusable:**By collecting a standardized set of mainly technical metadata e!DAL guarantees the long-term readability and usability of all published datasets. The schema is inspired by the DublinCore metadata format and meets community-established standards. Furthermore, a clear and easy procedure for license handling allows a suitable license to be assigned, which defines by whom and how the data can be used. They are available both on the content page of every data object as well as embedded in the HTML sources.

The concept of e!DAL to expose even dark [43] and semi-structured research data is also applied to metadata. They are divided into technical metadata, which are stored within e!DAL, and specific semantic metadata. This means a trade-off between a high volume of FAIR-enabled research data that contain only the technical metadata vs the opportunity to expose high-quality semantic metadata. Therefore, we propagate a 2-step procedure whose first step is to share and even preserve research data without semantic metadata that would otherwise tend to get moved into the attic of dark data. This is because there is frequently a discrepancy between community-accepted policies and practical resources for their execution in practice for data capture and the publication process. It is still a matter of resources and research data management culture to consider semantic data annotation as a reputational task within the scientific credit system. This has to be accompanied by research institute policies and data stewardship concepts. Nevertheless, until such general cultural change and its wide implementation in the research landscape, we aim minimally at exposing research data even with technical metadata only. The major goal of developing e!DAL is to provide a generic and data domain–agnostic infrastructure that could be set up and integrated easily. Therefore, the second phase of semantic metadata annotation must be anchored within the research organizations and their defined reviewers to take care that published datasets are in the scope of their repository and provide suitable metadata. This enables e!DAL to ensure FAIR data by providing technical metadata that can be used to mint DOIs and guarantee long-term preservation of research data. For example, the aforementioned PGP repository, which is hosted at the IPK Gatersleben, focuses among other things on plant phenotyping data. Therefore, the reviewers carefully check whether submitted datasets providing phenotypic data contain MIAPPE-compliant [15] metadata. A further challenge for scientists is to choose the suitable public and community-approved repository to share their data, assuming they have no recommendation, e.g., by journal publishers or research data management plans defined by the project. This is part of the review process for the PGP repository. If the appropriate repository for data submissions is available, the submission is rejected and the author is notified about suggested alternative places to deposit the data. Additional e!DAL-based repositories may focus on other domain-related policies and evaluate different aspects of submissions, which is a possibility that is baked into the design of the generic and open concept of the e!DAL software.

### e!DAL usage

Established in 2016, the PGP repository is the first productive repository based on the e!DAL infrastructure and a part of the service portfolio of the GCBN unit (German Crop BioGreenformatics Network) [25] of de.NBI (German Network for Bioinformatics Infrastructure) [44], which is the head of ELIXIR Germany. After >3 years of productive use, the PGP repository currently shares comprehensive, plant-related research datasets containing mainly genomic and phenomic information, but also metabolic datasets and software components and pipelines. Most of the datasets are part of a corresponding research paper and allow authors from IPK, but also from other institutes, to improve their manuscripts by enriching them with the underlying research data in a FAIR-compliant way. The overall download volume and large number of distinct user accesses show the high visibility of the provided datasets and the interest of the research community in this kind of research data.

The integration of the ELIXIR AAI into the login mechanism of the PGP Repository is a prime example that shows how established platforms can benefit from the ELIXIR network. The provided services contribute to the increase in visibility, to overcome the obstacles to the use of available infrastructures and to support FAIR-compliant access to research data. The support of the ELIXIR single sign-on service enables collaborators to use the PGP repository as a service to publish their research data. Furthermore, the ELIXIR AAI login is fully integrated into the e!DAL infrastructure software, which allows additional FAIR in-house repository instances to be set up following the presented I2D approach. Doing so in June 2018 a second repository based on the developed e!DAL infrastructure was established at the Forschungszentrum Jülich. Owing to the auto-configuring installation it was possible to run the system and provide the submission and review workflow with only a little effort in time. The integrated ELIXIR AAI login allows researchers from Jülich to use their existing institutional accounts. The complete infrastructure is hosted and maintained by the JPPC. The process of establishing further e!DAL-based repositories at the Julius-Kühn Institute and the Helmholtz Centre München is currently underway.

## Outlook

In this work, we showed the newly designed I2D concept for FAIR-compliant data publication by using in-house storage infrastructure and new features of the e!DAL platform. After several years of operating a productive instance of this infrastructure as the basis for the PGP repository, we recorded high numbers of accesses and downloads. Although researchers have more and more opportunities for sharing their research data with the community, the incentive to do so is still not high enough for some researchers [45]. In contrast to the common peer-reviewed publication in journals, it is not so easy to measure the impact of research data per se, because data citation is still not a common practice [46], although it is becoming more and more important and accepted [47]. This is not only a cultural problem but also a technical challenge and therefore an issue of practicability [48]. One of the first metrics to count data citations was the commercial Data Citation Index. But in the meanwhile some free and community-initiated projects such as Make Data Count have been developed. Furthermore, popular journals are starting to demand that authors cite their research data as data citations in their common reference list [49]. This facilitates measurement of its impact through a citation index and improves the visibility to readers, which in turn increases the general acceptance of research data as valuable scientific assets. In future we will investigate several approaches for counting data citations and getting more credit for publishing research data. We plan to integrate a generic and open source solution into the e!DAL infrastructure to show users comprehensive information about how their data is reused and referenced.

ORCID provides a widely accepted and used solution to unambiguously identify researchers. Its integration within the e!DAL infrastructure is intuitive and facilitates handling of multiple ORCIDs for comprehensive lists of authors. Besides the identification of persons, it can also be quite challenging to handle the diverse affiliations of research institutes, universities, or companies with a focus on different scientific topics. Some authors have multiple affiliations, from time to time organizations may be renamed, the official addressee may change owing to infrastructural developments, or it may happen that an institute will be closed. The Research Organization Registry (ROR) provides an open and sustainable approach, which is led by the community and supported by popular organizations like DataCite or Dryad. The concept of the ROR identifiers is very similar to the ORCIDs and allows all kinds of research organizations to be uniquely identified. Therefore, one of the next functional improvements for the e!DAL infrastructure will be the integration using the provided ROR API. This will cause some changes in the basic data structure, which however will result in a much easier and FAIRer way to handle author affiliations [50].

## Availability of Source Code and Requirements

- Project name: e!DAL (electronic Data Archive Library)
- Current version: 3.0.2
- Project homepage: https://edal.ipk-gatersleben.de
- Source Code Repository: https://bitbucket.org/ipk_bit_team/electronicdataarchivelibrary
- Operating system(s): platform independent
- Programming language: JVM based (Java 12+)
- JavaDoc: https://edal.ipk-gatersleben.de/javadoc
- Artifact repository: Maven Central (https://mvnrepository.com/artifact/de.ipk-gatersleben)
- License: GNU General Public License (GPL) Version 3 (https://www.gnu.org/licenses/gpl-3.0.html)
- BioTools: https://bio.tools/edal
- RRID:SCR_019017

The e!DAL project website provides comprehensive information that is relevant for users as well as developers. In addition, full Java documentation, further presentations, videos, and several code and use examples are presented. We changed the licence model of e!DAL to GNU General Public License (GPL) Version 3. This is aimed at maximizing the spread of the e!DAL infrastructure in the scientific community to foster FAIR principles of in-house stored data and to enable the incorporation of e!DAL into third-party software as well.

## Availability of Supporting Data and Materials

Snapshots of our code and other supporting data are openly available in the *GigaScience* repository, GigaDB [51].

## Abbreviations

AAI: Authentication and Authorization Infrastructure; API: application programming interface; de.NBI: German Network for Bioinformatics Infrastructure; DOI: Digital Object Identifier; DPPN: German Plant Phenotyping Network; EBI: European Bioinformatics Institute; ENA: European Nucleotide Archive; FAIR: Findable, Accessible, Interoperable, Reusable; GCBN: German Crop BioGreenformatics Network; I2D: Infrastructure-to-the-Data; IPK: Leibniz Institute of Plant Genetics and Crop Plant Research; JNLP: Java Network Launching Protocol; JPPC: Jülich Plant Phenotyping Center; JRE: Java Runtime Environment; JSON-LD: JavaScript Object Notation for Linked Data; MIAPPE: Minimum Information About a Plant Phenotyping Experiment; NCBI: National Center for Biotechnology Information; OAI-PMH: Open Archives Initiative Protocol for Metadata Harvesting; ORCID: Open Researcher and Contributor ID; PGP: Plant Genomics and Phenomics Research Data Repository; PGR: Plant Genetic Resources; REST: representational state transfer; RIA: Rich Internet Application; RMI: Remote Method Invocation; ROR: Research Organization Registry; URN: Uniform Resource Name.

## Competing Interests

The authors declare that they have no competing interests.

## Funding

This work was supported by the German Federal Ministry of Education and Research (BMBF) in the frame of the projects German-Plant-Phenotyping Network - DPPN (FKZ 031A053), Modernste Virtualitäts-und erweiterte Realitäts-Verfahren für den Zyklus von Samen zu Samen - AVATARS (FKZ 031B0770A), and German Network for Bioinformatics Infrastructure - de.NBI (FKZ 031A536A).

## Authors' Contributions

Conceptualization: D.A., M.L.

Software: D.A., P.K.

Investigation: D.A.

Supervision: M.L.

Writing original draft: D.A., M.L.

Writing review and editing: All authors

Funding acquisition: U.S., A.J., M.L.

## Supplementary Material

giaa107_GIGA-D-20-00181_Original_Submission

giaa107_GIGA-D-20-00181_Revision_1

giaa107_GIGA-D-20-00181_Revision_2

giaa107_GIGA-D-20-00181_Revision_3

giaa107_Response_to_Reviewer_Comments_Original_Submission

giaa107_Response_to_Reviewer_Comments_Revision_1

giaa107_Response_to_Reviewer_Comments_Revision_2

giaa107_Reviewer_1_Report_Original_Submission Noah Fahlgren -- 8/11/2020 Reviewed

giaa107_Reviewer_2_Report_Original_Submission Guy Cochrane, PhD -- 8/15/2020 Reviewed

giaa107_Reviewer_3_Report_Original_Submission Cyril Pommier -- 8/20/2020 Reviewed
