# Supplementary material for: The on-premise data sharing infrastructure e!DAL: Foster FAIR data for faster data acquisition
Source: Gigascience. 2020 Oct 22;9(10):giaa107. doi: 10.1093/gigascience/giaa107 (PMC7580168; doi:10.1093/gigascience/giaa107)
Supplement: giaa107_GIGA-D-20-00181_Revision_1 [file giaa107_giga-d-20-00181_revision_1.pdf]

GigaScience

The on-premise data sharing infrastructure e!DAL: Foster FAIR data for faster data acquisition

--Manuscript Draft--

**Manuscript Number:**	GIGA-D-20-00181R1
**Full Title:**	The on-premise data sharing infrastructure e!DAL: Foster FAIR data for faster data acquisition
**Article Type:**	Technical Note
**Funding Information:**	Bundesministerium für Bildung und Forschung (031A053) — Dr. Daniel Arend<br>Bundesministerium für Bildung und Forschung (031B0770A) — Mr. Patrick König<br>Bundesministerium für Bildung und Forschung (031A536A) — Dr. Daniel Arend
**Abstract:**	The FAIR data principle as a commitment to support long-term research data management is widely accepted in the scientific community. Although the ELIXIR Core Data Resources and other established infrastructures provide comprehensive and long-term stable services and platforms for FAIR data management, a notifiable number of research data is still hidden or under risk of getting lost. Currently, high-throughput plant genomics and phenomics technologies are producing research data in abundance, the storage of which is not covered by established core databases. This concerns the data volume, e.g. time series of images or high-resolution hyper-spectral data, the quality of data formatting and annotation, e.g. with regard to structure and annotation specifications of core databases, uncovered data domains or organizational constraints to not primarily store data outside the institional boundaries. In order to share these potentially "gray'" data in a FAIR way and master these challenges the ELIXIR Germany/de.NBI service Plant Genomic and Phenomics Research Data Repository (PGP) implements a "bring the infrastructure to the data" approach which allows to keep the research data in place and wrap them by a FAIR-aware software infrastructure. This article presents new features of the PGP infrastructure as a best practice on how to easily set up FAIR-compliant and intuitive research data services. Furthermore, the integration of the ELIXIR Authentication and Authorization Infrastructure (AAI) and data discovery services are introduced as means to lower technical barriers and to increase the visibility of research data.
**Corresponding Author:**	Daniel Arend, Ph.D.<br>Leibniz-Institut für Pflanzengenetik und Kulturpflanzenforschung<br>Seeland OT Gatersleben, SACHSEN-ANHALT GERMANY
**Corresponding Author Secondary Information:**	
**Corresponding Author's Institution:**	Leibniz-Institut für Pflanzengenetik und Kulturpflanzenforschung
**Corresponding Author's Secondary Institution:**	
**First Author:**	Daniel Arend
**First Author Secondary Information:**	
**Order of Authors:**	Daniel Arend<br>Patrick König<br>Astrid Junker<br>Uwe Scholz<br>Matthias Lange

Powered by Editorial Manager® and ProduXion Manager® from Aries Systems Corporation

**Order of Authors Secondary Information:**	
**Response to Reviewers:**	Comments and responses to reviewer comments<br><br>Reviewer 1:<br><br>Summary<br>-----------<br>The manuscript "The on-premise data sharing infrastructure e!DAL: Foster FAIR data for faster data acquisition" by Arend and colleagues provides an overview of updates and enhancements to the e!DAL on-premise data-sharing platform. The authors address common issues with scientific data sharing using centralized repositories: 1) data (potentially a large quantity) needs to be transferred from the owner to a repository; 2) data often need to repackaged in various ways to fit the expectations of the repository; 3) dataset metadata needs to potentially be tailored to each repository; 4) costs associated with central repositories (direct or indirect, e.g. training); and 5) lack of central repositories for some data types (e.g. imaging and phenotype data). To address these issues, e!DAL provides the infrastructure to achieve data sharing using FAIR principles using on-premise data infrastructure. e!DAL provides a data publication layer on top of a data repository that interfaces with existing publication and search engine infrastructure to make shared datasets FAIR.<br><br>Strengths<br>-----------<br>* The manuscript is well-written and succinctly describes the rationale for and improvements of e!DAL.<br>* The figures effectively summarize the major features of e!DAL and the results of its usage.<br><br>Weaknesses<br>--------------<br>* None<br><br>We want to thank the reviewer for his positive feedback and effort to review our manuscript.<br><br>Suggestions for improvement<br>---------------------------------<br>My understanding is that GPL licenses require an exact copy of the license be distributed with the software: https://www.gnu.org/licenses/gpl-faq.html#WhyMustIInclude. The current license file(s) in e!DAL only states that GPLv3 applies but there does not seem to be a copy of GPLv3 itself.<br><br>Thank you for the hint, we added a full version of the license text to the license file in the Bitbucket repository of e!DAL.<br>https://bitbucket.org/ipk_bit_team/electronicdataarchivelibrary/src/master/LICENCE.md<br><br>The manuscript discusses that "By collecting a standardized set of mainly technical metadata e!DAL guarantees a long-term readability and usability of all published datasets." This is important for making datasets findable, but there is an additional layer often required to use data, which is documentation of what the data is, how it was collected, how it is structured, etc. While not in the scope of the current work, could the authors potentially discuss in the outlook section whether they see this type of metadata or documentation being a component of e!DAL? In particular, I am thinking of metadata ontologies like MIAPPE, MIxS, etc.<br><br>You are right, of course semantic metadata is an important point. But the e!DAL concept to expose even "grey" and semi-structured research data is applied to the metadata too. Here we differentiate between generic, technical metadata, which are stored within e!DAL and specific semantic metadata. We are aware of the trade-off to make as much research data as possible FAIR and expose high quality semantic metadata. We think it is beneficial to expose datasets even without mandatory semantic metadata. Because it is still a resource challenge for scientists to annotate research data accordingly. Due to the strong heterogeneity and diversity of research data and semantic metadata schema as well technical challenges, this has to be

accompanied by institutional policies and Data Stewards. However, until a general cultural change and its wide implementation in the research landscape, we aim minimally at exposing research data even with technical metadata only. The major goal of the development was providing a generic infrastructure that could be set-up and integrated easily. Supporting specific semantic metadata schemas like MIAPPE would need massive effort and institute specific adaptation for every instance. Therefore we think it is the task for the hosting institute or rather the specific reviewers of ever instance to take care that every dataset that they accept is in the scope of the certain instance and provide suitable semantical metadata, while e!DAL takes care that the technical metadata, which are needed to guarantee long-term readability and which are necessary to assign a DOI, are provided. For example, the mentioned PGP repository, which is hosted at the IPK Gatersleben focuses among others on plant phenotypic data. Therefore our reviewers carefully check if every submitted dataset providing phenotypic data contains a MIAPPE compliant metadata description. Other e!DAL based repositories may have a focus on
different data domains and therefore evaluate every submission
differently.

Reviewer 2:

This manuscript provides a clear update on the e!DAL software package for the operation of local data repository infrastructure. Following the "Infrastructure to data (I2D)" model, the tool is available for reuse and is currently operational in two institutions (e.g. operating the "Plant Genomics and Phenomics Data Repository (PGP)" at IPK-Gatersleben) with a further two currently installing it. The manuscript provides details on some appropriate updates and additions that include enhanced data ingress performance, addition of the ORCID identifier system for contributor authentication, the ELIXIR AAI, JSON-LD presentation format, use of Gradle build/deployment infrastructure and a choice of two data upload/submission routes - full application and web application.

We want to thank the reviewer for his positive feedback and effort to review our manuscript

e!DAL and its implementations (especially PGP, which could be considered the "reference" implementation), lie within the ELIXIR ecosystem. As the authors note, e!DAL occupies a niche in this ecosystem that is as yet unfilled for many of the data types associated with plant phenomics. However, it also has in scope data of types that can be handled by other elements of the ecosystem, such as the ELIXIR Core Data Resources. These include, for example, genomics databases (see a data set of relevance for this, for example, at https://doi.ipk-gatersleben.de/DOI/1c5dc9c8-0b38-4b2b-93d3-993272532cb1/711ad917-d85b-4e08-b883-8af94ae215b0/2). I recommend that the authors address this issue in the system and the manuscript: how does the system ensure that incoming data sets that include data types appropriate for deposition elsewhere in ELIXIR are appropriately routed and linked from the system?

Yes you are right, the mentioned dataset would also fit into established genomic databases. As we also mention in the following question, the reviewers are mainly responsible to take care about the data quality and that the dataset fits into the scope

Powered by Editorial Manager® and ProduXion Manager® from Aries Systems Corporation

of the repository. In the case of the mentioned dataset the reason for its deposition into the e!DAL-PGP repository was due to the less strict recommendation policy in Nature for the publication of miRNA loci and the necessity of an ad-hoc publication of this data set to meet the submission deadlines for the paper (https://dx.doi.org/10.1038/nature22043). However, we are aware that choosing the right public repository is an important issue. We consider this in the review process and always try to recommend the most appropriate repository for data submission.

While promoting FAIR principles, it is not clear from the manuscript how e!DAL supports compliance for data sets with community data standards. I would have expected reference in the text, for example, to ELIXIR-related data standards for plant sciences such as MIAPPE and the Breeding API (https://elixir-europe.org/communities/plant-sciences).

Of course you are right it is important to support and push established community standards, but this is very difficult due to the strong heterogeneity of research data and not objective of the generic e!DAL software, because it is data domain agnostic. This is rather a task of the data submitters to take care that he uses established standards and metadata schema to provide FAIR research data. Nevertheless, we agree this should be the task of the reviewers of the specific e!DAL based repositories. However, we see a gap between FAIR culture and its implementation in projects by dedicated resources and monitored processes whereas every dataset needs to be checked in respect of data and metadata quality. As argued before, it is a matter of policies and resources at the site of data producer and reviewer, too.

e!DAL takes the I2D model in which distinct repositories operate at institutional level and are connected through the DOI system of identifiers. The authors lay out some but not all of the features of such a model. While they correctly declare that in the event that an e!DAL repository is removed from service, metadata relating to its content will remain in the DataCite system, this is far from optimal as the data themselves will have been lost. In the I2D model, what mechanisms exist, or could be put in place, to protect against such loss?

That is a very good analysis of the described I2D model. There is no out-of-the-box solution within the e!DAL infrastructure software, but due to the I2D concept is the task of the hosting institute to protect against data loss by protecting the hardware components on which the e!DAL instance is running. For example the mentioned e!DAL-PGP repository, which is hosted at the IPK Gatersleben, is using a powerful HSM (hierarchical storage management) infrastructure, which backups the stored datasets and protects them in case of hardware failures or other issues. In addition, the other instances running at the research centre Jülich or at the JKI are using similar backup solutions based on their local, institutional hardware infrastructure. Maybe in the future it is worth thinking about an embedded support within the e!DAL software like providing an option to set-up the infrastructure on distributed systems to help the installing institute to protect against data loss, but this could be quite challenging and would also increase the needed effort to set-up repositories based on the e!DAL.

Powered by Editorial Manager® and ProduXion Manager® from Aries Systems Corporation

Reviewer 3:

This paper describe a sustainable data repository solution that provides an intermediate between the most important international data repositories and non-sustainable project databases. It provides an alternative to Research institutions dataverses or dspace with the promise of reduced deployment costs. This paper therefore discuss a very important question.

The alternate software solutions are fairly presented and the advantages of e!DAL are correctly discussed.

The paper is well written and organized. Some modifications are proposed below.

The software and data repositories are publicly accessible and the software is under GPLv3 open source license. The source code repository is missing a LICENSE file though.

The technical details are clearly introduced and discussed with sufficient information. The reuse of the code is well documented but I haven't tested it.

Therefore the proposition is to request Minor revisions before publishing this quality article.

We want to thank the reviewer for his very detailed feedback and effort to review our manuscript.

Remarks:

Some references lack DOI Nature genetics, New Phytologists, …)

Thank you for the hint, we checked all references again and added missing DOIs

DOI minting is not discussed, it could be a plus as getting DOI as a cost for an organization.

We understand the question in two aspects. First situation is that an external user submits his data to an existing e!DAL repository, like the PGP repository at the IPK. Then of course he can get his DOI for free, because the IPK as a hosting institute is registered as a data centre in the DataCite consortium and pays for DOI minting. On the other side if another institution decided to use e!DAL to set-up his own instance then they have to take care to become a DataCite member to get an own account for their repository to mint DOIs. But anyway our experience is that for many institutes in the past it was not a major challenge to get a DataCite account or to pay for the DOI minting, but it was missing a suitable infrastructure software to organize their datasets and to assign a DOI. And with e!DAL they have an easy and generic solution for this problem.

A GPLv3 license file should be added in the eDALE code repository.

Thank you for the hint, we added a full version of the license text to the license file in the Bitbucket repository of e!DAL.
https://bitbucket.org/ipk_bit_team/electronicdataarchivelibrary/src/master/LICENCE.md

-- Abstract-- :
« the storage of which is not covered by established core databases " : There are established databases, some are Elixir Core for Genomic and others only established for Phenomics.

Powered by Editorial Manager® and ProduXion Manager® from Aries Systems Corporation

Yes, you are right this was poorly formulated. We modified this section in the abstract to make this point as well as the following one more clear.

"Due to its high volume and strong heterogeneity, resulting in missing infrastructures" : this sentence should be clarified.

See previous answer.

"ELIXIR AAI » : the purpose of this service isn't obvious for non Elixir reader.

Yes that is absolutely true, due to the word limit in the abstract we have to avoid to go into detail here, but we added the full form of AAI ('Authentication and Authorization Infrastructure'), because this should give an idea of the purpose even for non ELIXIR readers.

"as means tolower « : typo

We corrected the typo.

--Introduction--
"general purpose data repositories, e.g. figshare [6], Zenodo [7] and Dryad [8] " : FAIRDOM is another repository that might be worth mentioning.

Yes, thanks for the idea, we added FAIRDOM.

"(iii) institutional ": research institute might better reflect the authors intention

Yes that is correct, we reformulated that point.

", e.g. the EBI and NCBI core data resources, Bioinformaticians are charged and trained ": the end of this sentence should be clarified

We changed the sentence a little bit to make clear that bioinformaticians are often necessary due to the diverse and specific submission requirement for some repositories.

"or the preparation of ISA-TAB compatible data submission for plant phenotyping data [14, 15]. ": MIAPPE paper: this is the first one, two others have been published since 2015. The latest should be the most accurate.

That is correct, we replaced the reference with the latest article: https://doi.org/10.1111/nph.16544

"Alternatively, project-related or institutional data repositories could be set up. " Can Research institution repositories and project databases be really placed at the same level ?

Yes, you are right this is poorly formulated. We changed the sentence.

"This finally enables the assignment of DOIs with a minimal set of metadata to in-house stored data and its approved FAIR refer- encing by journals or data lookup services. ": it would be worth describing a little be more the metadata (Dublin core minimal dataset or a more extensive list of minimal information about… MIA*)

The metadata are technical and administrative metadata and based on the DublinCore. We added a short phrase to clarify this. Details are already described in the first e!DAL publication, which is also cited in the same section (http://dx.doi.org/10.1186/1471-2105-15-214). Therefore we have not gone into detail here.

"Approximately seven million crop accessions « : the term accession should be describe a little bit more for new readers (eg PGR accession)

Thanks for the hint, we added an additional sentence and reference, as well as

Powered by Editorial Manager® and ProduXion Manager® from Aries Systems Corporation

reformulated the phrase.

"that do not fit into classical databases due to their volume " : What are precisely those classical databases ? It would be good to refer to the three categories of the first paragraph.

We reformulated the sentence to clarify this and refer to the previous mentioned types in the first paragraph.

"This experience and the adoption as a service in the European life-sciences Infrastructure for biological Information ELIXIR [23] " : the words "adoption as a service" would need clarification or rephrasing.

Yes, this is correct, “adoption as a service” seems to be a quite strong wording. We rephrase the sentence and also add a link to the list of ELIXIR services.

-- Related Work --

"Most of them evolved over many years and they are widely accepted by the research community [24] " the reference 24 is a bit old (2010) if possible a newer one would be a plus.

Thanks for the hint, we added a newer publication: https://doi.org/10.1038/nplants.2017.86

"Usually also research journals and other publishers request data sharing using these established domain- specific databases. " They require the use of sustainable repositories which can be found using means described in the next sentence. This should be clarified.

Thanks for the comment, we fully rephrase the sentence to make the point more clear.

"[9] like BRENDA [30] or SILVA [31] « those are deposition databases I assume. This could be clarified.

Yes they are ELIXIR deposition databases. We added this fact to the sentence.

-- Infrastructure—
"Figure 2" : The term edal project is a little bit confusing: is it a repository, a software project, all of that ? edal website only could be clearer. Or possibly using software project instead of project alone. This screenshot might need updating.

Yes, you are right this is poorly formulated. Sure, e!DAL is software, but it was of course initially developed in the frame of a project. That’s why we named it “project website”, but yes this can be confusing. We completely re-formulated the figure caption.

-- Improvements—
"implementation of the e!DAL infrastructure was necessary. " : it is not clear that the following paragraph describe those changes.

Yes, you are right this is ambiguous formulated. We added an additional sentence to make this clear.

-- New Features—
Has Dublin core been cited yet ?
"To add the possibility for assigning an ORCID to every data creator or contributor in the e!DAL infrastructure, the PERSON data type in the metadata schema " : is this the person from an internal metadata scheme or dublinCore/Schemas.org scheme ?

	Yes as you assumed this is the metadata attribute from the internal metadata schema, which is inspired by the DublinCore Schema. But in contrast to the DublinCore schema, which does not prescribe any data type for describing the specific attributes, e!DAL bind attributes to dedicated data types. This enables data validity and features the GUI to support users to enter valid metadata elements to describe his datasets. So e.g. the mentioned PERSON data type can be used to set the metadata attributes “Author” or “Contributor”. We do not go into detail here, because this was already a part of the first version of e!DAL and our first publication (http://dx.doi.org/10.1186/1471-2105-15-214). We here just focused on the comprehensive update to link the PERSON attribute with the ORCID API. To make this clearer for the reader, we added here again the reference to the first publication and slightly changed the sentence.<br><br>"The new e!DAL login module follows the OAuth protocol [40] to authenticate users over the ELIXIR AAI and automatically receive their email address, ": the email address is used as a technical ID of the authenticated user, no ?<br><br>Yes you are right, from the conceptual perspective the email address of the user will be used for two aspects. First of all for the communication within the embedded data review process of e!DAL, so that the submitting user gets information about the status of his submission and also receives the final DOI via email. And secondly it is also a kind of “technical” or “internal” ID to authenticate the user within the e!DAL infrastructure.<br>The second point was from our perspective not so interesting for the reader, but we added an additional sentence into the manuscript to make this clearer.<br><br>-- OS specific executables -<br>Some minor grammar improvement or sentence clarity in this section.<br>"ava Network Launching Proto- col (JNLP) ": precise that it is the basis of java web start<br><br>Thanks for this hint, we corrected this and added a clarification.<br><br>--Results—<br>"Accessible" : the description is true but should be applied to eDale, it is rather general in its current form.<br><br>Yes you are right, we reformulated that section to apply this more concrete to e!DAL.<br><br>--e!DAL Usage—<br>"After more than three years of productive usage, the PGP " production?<br>The last paragraph might be slightly redundant with previous statements.<br><br>Yes you are right some points were slightly redundant, we fixed this and changed some sentences in this paragraph.
**Additional Information:**	
**Question**	**Response**
Are you submitting this manuscript to a special series or article collection?	No
**Experimental design and statistics**<br><br>Full details of the experimental design and statistical methods used should be given in the Methods section, as detailed in our Minimum Standards Reporting Checklist. Information essential to interpreting the data presented should be made available	Yes

in the figure legends.<br><br>Have you included all the information requested in your manuscript?	
**Resources**<br><br>A description of all resources used, including antibodies, cell lines, animals and software tools, with enough information to allow them to be uniquely identified, should be included in the Methods section. Authors are strongly encouraged to cite Research Resource Identifiers (RRIDs) for antibodies, model organisms and tools, where possible.<br><br>Have you included the information requested as detailed in our Minimum Standards Reporting Checklist?	Yes
**Availability of data and materials**<br><br>All datasets and code on which the conclusions of the paper rely must be either included in your submission or deposited in publicly available repositories (where available and ethically appropriate), referencing such data using a unique identifier in the references and in the "Availability of Data and Materials" section of your manuscript.<br><br>Have you have met the above requirement as detailed in our Minimum Standards Reporting Checklist?	Yes

Powered by Editorial Manager® and ProduXion Manager® from Aries Systems Corporation

Manuscript

Click here to access/download;LaTeX - Main Document (TeX file);gigascience.tex

OXFORD

Placeholder for journal logo gigascience-logo.pdf

GigaScience, 2018, 1–10

doi: xx.xxxx/xxxx
Manuscript in Preparation
Technical Note

TECHNICAL NOTE

The on-premise data sharing infrastructure e!DAL: Foster FAIR data for faster data acquisition

Daniel Arend[1*], Patrick König[1], Astrid Junker[1], Uwe Scholz[1] and Matthias Lange[1]

[1]Leibniz Institute of Plant Genetics and Crop Plant Research (IPK) Gatersleben

[*]arendd@ipk-gatersleben.de

Abstract

The FAIR data principle as a commitment to support long-term research data management is widely accepted in the scientific community. Although the ELIXIR Core Data Resources and other established infrastructures provide comprehensive and long-term stable services and platforms for FAIR data management, a notifiable number of research data is still hidden or under risk of getting lost. Currently, high-throughput plant genomics and phenomics technologies are producing research data in abundance, the storage of which is not covered by established core databases. This concerns the data volume, e.g. time series of images or high-resolution hyper-spectral data, the quality of data formatting and annotation, e.g. with regard to structure and annotation specifications of core databases, uncovered data domains or organizational constraints to not primarily store data outside the institional boundaries. In order to share these potentially "gray" data in a FAIR way and master these challenges the ELIXIR Germany/de.NBI service *Plant Genomic and Phenomics Research Data Repository (PGP)* implements a "bring the infrastructure to the data" approach which allows to keep the research data in place and wrap them by a FAIR-aware software infrastructure. This article presents new features of the PGP infrastructure as a best practice on how to easily set up FAIR-compliant and intuitive research data services. Furthermore, the integration of the ELIXIR Authentication and Authorization Infrastructure (AAI) and data discovery services are introduced as means to lower technical barriers and to increase the visibility of research data.

Key words: research data management; FAIR principles; digital object identifier; plant genomics and phenomics

Introduction

The FAIR (Findability, Accessibility, Interoperability, and Reusability) principles, drafted by the FORCE11 workgroup in 2015 [1] and published in 2016 by Wilkinson et al. [2] are widely accepted and are increasingly adopted in the management policies of research data. The scientific community is showing a rising awareness of the scientific value of reusable research data.This has already resulted in FAIR principles being formally accepted in several data management guidelines, e.g. in the Horizon2020 program [3] of the European Commission, and integrated into the research funding policy [4, 5]. Its technical implementation is supported by data repositories, which store and share research data in a FAIR manner. Those can be classified into (i) general purpose data repositories, e.g. figshare [6], Zenodo [7], Dryad [8] and FAIRDOM [9], (ii) core data deposition databases, i.e. ELIXIR deposition databases for life science data [10], NCBI database resources [11] and (iii) specific databases and repositories hosted by research institutes. All have in common that the research data has to be transferred by its owner from the place of data generation to these repositories. This involves considerable effort for data compilation, cleansing, homogenisation, metadata enrichment, formatting and upload. As a result, the published datasets are condensed and generally limited to insufficiently documented supplement material for publications in scientific journals. In the case that data should be submitted to database systems, e.g. the EBI and NCBI core data resources, Bioinformaticians are charged and trained to meet the specifict submission requirements and support biologists. Examples are the preparation of data for

Compiled on: September 9, 2020.
Draft manuscript prepared by the author.

submission to the EBI ENA archive [12, 13], the European Variation Archive (EVA) [14] or the preparation of ISA-TAB compatible data submission for plant phenotyping data [15, 16]. Alternatively, institutes could set up project-related data repositories. This in turn requires skilled technicians and computer scientists as well as long-term access to appropriate network and storage infrastructure. Such repositories frequently have a short life time, whether due to staff fluctuation, long-term maintenance costs and resource consumption. Another reason may be that the repository's niche is too specific to attract substantial data volume, which in turn strongly depends on policies and cost-benefit considerations.

Thus, there is a need for an additional class of repositories that support the data sharing for this class of research data by moving the infrastructure to the data. The concept is to apply an on-premise, infrastructure-to-the-data (I2D) principle. The basic idea of the I2D approach is shown in Figure 1. In contrast to the conventional data publication pipelines to journal accepted databases, which usually involves a time-consuming data upload to an external platform and possibly additional costs depending on the required storage space, the underlying e!DAL software [17] encapsulates an existing storage infrastructure by a data publication layer. This layer is a broker to the DataCite [18] data publication service agent and provides an API and a tooling infrastructure for data submission, DOI delivery, reporting and data quality reviewing. This finally enables the assignment of DOIs with a minimal set of technical metadata, which are based on the DublinCore, to in-house stored data and its approved FAIR referencing by journals or data lookup services.

As proof of concept, the Plant Genomics and Phenomics Data Repository (PGP) was implemented [19] to publish digital plant genetic resources (PGR) [20] according to the FAIR principles. PGRs are the basis of food security and consist of diversity of seeds and planting materials of modern cultivars and crop wild relatives [21]. Approximately seven million PGR accessions are conserved in genebank collections worldwide. The valorisation of PGRs through genotyping and phenotyping is of special focus in the public and private sectors [22, 23]. The data management of digital PGRs is identified as one of the most important challenges for a long-term strategy to enhance the productivity, sustainability and resilience of crop varieties and agricultural systems. In contrast to successful studies on genomics-assisted genebank management and the utilization of germplasm collections [22], the special focus of the PGP repository is the publication of buckets of research data that do not fit into general purpose sharing platform or core data deposition databases due to their volume, objective, structure or incomplete analysis. Examples are primary data from imaging, field phenotyping, SNP matrices, 3D-plant models, metabolite screenings and environmental sensor data. The experience gained during the four-year operation of the repository has led to a growing acceptance of this approach for the publication of digital PGRs collected in the context of the German Federal ex situ Genebank of Agricultural and Horticultural Crop Species [24]. This experience and the adding to the *list of service* in the European life-sciences Infrastructure for biological Information ELIXIR [25] resulted in novel features, which were implemented with the aim of further improving its acceptance and enabling increased sharing of digital PGRs. After an update to the state of the art, the new features of the e!DAL data sharing software and its application for the publication of digital PGRs will be explained.

Related Work

Just like we have many different data types from several domains, there are also a variety of domain-specific archives and information systems. Most of them evolved over many years and they are widely accepted by the research community [26], *ENA* for genomic data [27], *UniProt* for protein data [28], *PRIDE* for proteome data [29], *BioModels* for system biology data [30] and many more. As a guideline, research journals and other publishers require the sustainable publication of data according to FAIR criteria. For this purpose, established domain specific databases or the use of long term committed data repositories is recommended. In order to not get lost in the diversity of archives, there are several registries like *re3data.org* or FAIR-sharing.org, as well as consortia like GFBio which collect and categorize repositories to help researchers finding the suitable storage for their data.

Infrastructure programs like the European Open Science Cloud (EOSC) [4], and the European life-sciences Infrastructure for biological Information (ELIXIR) [31] coordinate maintenance and interoperability of research data repositories as federated services by member organisations and hosting institutions. Furthermore, the ELIXIR organisation has the aim to establish a stable and sustainable infrastructure for biological information. In doing so, they define important core resources and deposition databases as a support of the research community [10] like *BRENDA* [32] or *SILVA* [33]. Most of these systems accept only very specific datasets and require specialised metadata based on schemes that have been improved by the community over years. Unfortunately, there are several, mostly relatively new data types, e.g. plant phenotypic data, which currently do not fit into existing databases, mainly because of their strong heterogeneity and high volume. Public data sharing ser-

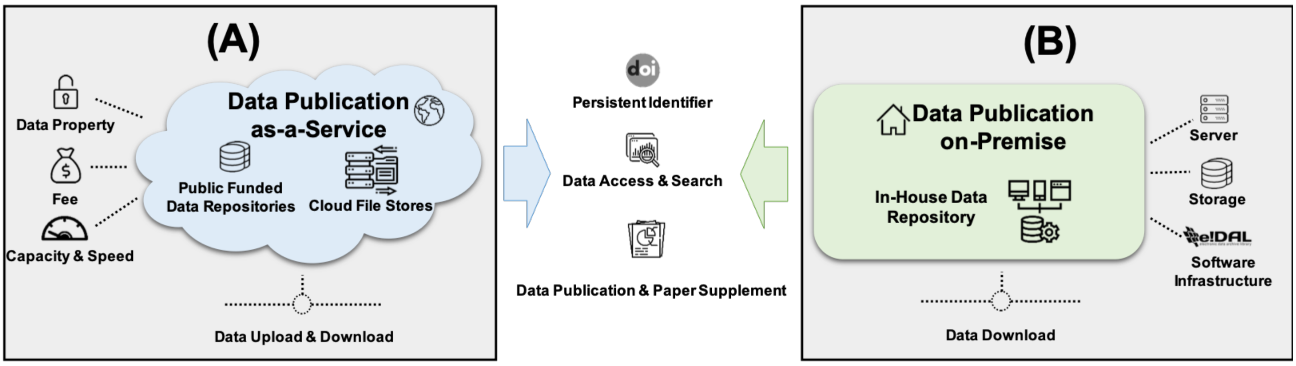

Figure 1. Data-Publication-as-a-Service vs. Data-Publication-on-Premise: Both services feature FAIR data publication, but differ in costs. The as-a-service model (A) costs fee, delegation of data property control and face capacity limits in storage and data upload. The on-premise model (B) keep data in-house, but requires the availability of server and storage hardware as well as the installation of the e!DAL software.

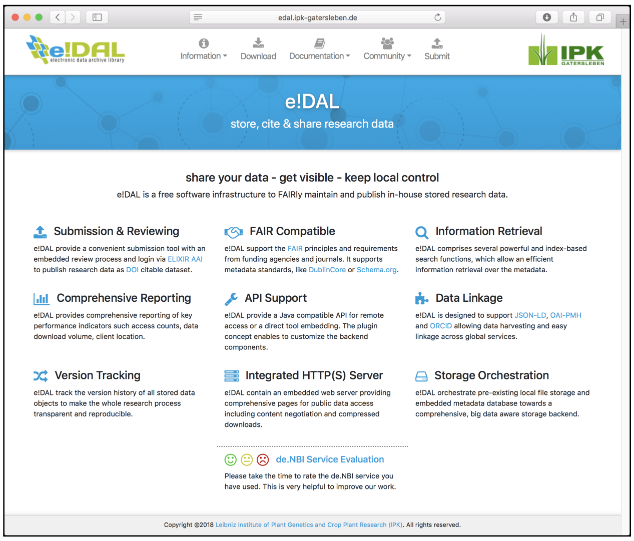

Figure 2. Screenshot of the e!DAL website. Here a very detailed description on how to use the e!DAL infrastructure software as well as comprehensive examples for users and developers are provided. Furthermore, some video tutorials and presentation recordings are available to lower the initial barriers to dig into the e!DAL infrastructure.

vices like *figshare* or *DRYAD* provide an alternative solution for publishing these datasets. They are easy to use and have a comprehensive functionality like supporting version controlling and the assigning of persistent identifiers. One important deficiency of such services is the limited free space, which is usually enough for sharing some reduced graphics or aggregated tables, but not for storing large datasets. Furthermore, the establishment and configuration of an own in-house infrastructure based on existing software packages like *CKAN* or *Dataverse* could overcome this shortcoming, but they require quite a lot of technical prerequisites and know-how.

Infrastructure

To lower the technical barriers and minimize the effort for scientists to archive and share their research data, we developed the generic e!DAL software infrastructure [17]. The usual "Data Publication as-a-Service" procedure includes the transfer of selected datasets to external databases and storage infrastructures after data generation and analysis. In this way research data can be referenced in a future research publication, as show on the left side of Figure 1 (A). In contrast the e!DAL infrastructure provide a "Data Publication on-Premise" approach which enables the publication of locally stored high voluminous research data through the assignment of widely accepted and long-term stable Digital Object Identifiers (DOIs). This is illustrated on the right side of Figure 1 (B). Using DOIs for referencing provides multiple advantages for sharing and accessing research data. Beside adding them as supplements to a research article they can also be the basis for a comprehensive data paper [34]. Furthermore, the well connected infrastructure of the DataCite consortia strongly increase the visibility of the research data assigned with a DOI. It is automatically linked with the ORCID account of the authors, can be found via the DataCite Search and other common search engines or can be harvested via OAI-PMH interface.

Based on the e!DAL infrastructure, the Leibniz Institute of Plant Genetics and Crop Plant Research (IPK) Gatersleben and the German Plant Phenotyping Network (DPPN) jointly have initiated the Plant Genomics and Phenomics Research Data Repository (PGP) [19] as a powerful infrastructure for the publication of comprehensive plant genomics and phenomics research data. The repository covers in particular cross-domain datasets, which are not being published in public repositories for reasons of data volume or data domain, such as phenotyping images, genotyping data, visualizations of morphological models, data from mass spectrometry as well as software and related documents. Doing so, PGP currently provides 200 data records, which can be referenced via DOIs and are annotated with technical metadata. These records comprise more than 1.4 million files with an overall volume of over 2.6 terabytes (see Figure 3). To ensure data discoverability, PGP provides landing pages with JSON-LD formatted metadata and is therefore discoverable through data web crawler services, which follow the schema.org recommendations, such as Google, Microsoft, Yandex etc. Furthermore, e!DAL implements the OAI-PMH Protocol for Metadata Harvesting from Open Archives Initiative. To support scientists to disseminate their research data the PGP infrastructure is accepted as institutional repository for the Scientific Data (Nature Publishing Group) and the GigaScience (Oxford Academic) Journals and is registered in re3data.org, FAIRsharing.org, OpenAIRE and DataCite.

The benefits of this wide support of data discovery enabling technologies and data publication in general is proven by the steadily increasing number of dataset accesses. By June 2020, PGP delivered 300 terabytes of data and the provided datasets have been accessed by 100,000 unique clients.

Improvements

The following section will sum up the main enhancements and updates of the e!DAL infrastructure which comprise new general features, comprehensive changes of several frontend components and important performance improvements. Furthermore, an extensive update due to the latest changes in the Java programming languages and an improved build and deployment process is described.

Performance

After releasing the first productive version of the PGP repository in 2015, we received many diverse data submissions from several research domains and with very heterogeneous data files. Since then we recognized that the e!DAL infrastructure software scales very well and is able to handle millions of data files, which confirms previous calculations and performance tests [17]. But furthermore it became apparent that sometimes the performance decreases, e.g. for uploading comprehensive datasets with several hundred thousands of small files. Since this is a very common case, e.g. for plant phenotyping datasets, an improvement of the implementation of the e!DAL infrastructure was necessary. Some major performance improvements are described subsequently.

One important feature of e!DAL is the automatic calculation of several essential technical metadata, like the MIME-Type, the data volume or the checksum of every file when storing new datasets. This is convenient , because the user does not need to provide these information on his/her own, but of course these computations are a resource- and runtime-intensive process. Therefore, the functionality to determine the previously mentioned technical metadata and the procedure to transfer the actual binary data have been improved towards a parallel processing of multiple files. This results in a better performance especially on today's multi-core systems. Furthermore, we optimized several settings for the streaming buffer size and the remote transfer to improve the memory usage and the upload performance for the case of numerous small files. Additionally

the checksum calculation was updated to use the more collision resistant SHA-256 algorithm, instead of the older and unsecure MD5 function.

New Features

The previous version of the e!DAL infrastructure already fulfilled several recommendations of the FAIR data principles, such as the support of standardized metadata based on the DublinCore schema or the provision of persistent DOIs for accessing and referencing of research datasets. The e!DAL infrastructure has been further updated to optimize the usability and the general user experience. Additional features where implemented to increase the visibility of published data and the acceptance of the infrastructure, which in the end also led to be even more FAIR compliant. Thereby the roadmap for scholarly data repositories [35] was taken into account. Subsequently, the most important extensions are described.

ORCID

To efficiently find and access specific research data files across millions of datasets, persistent identifiers like DOIs or URNs are very helpful and well established. But nevertheless, the research community is also quite large, and sometimes it is very difficult to distinguish data authors because of similar names or to identify the same researcher after he changes his affiliation. With the *Open Researcher and Contributor ID* (ORCID) there is an easy and persistent solution to uniquely identify authors and to solve issues with name ambiguity [36]. An important advantage is the interdisciplinarity, because ORCID is used across nearly all research domains and organizations, e.g. at the mid of 2019 in Germany there were already 150.000 ORCIDs registered [37]. By linking authors with publications, affiliations or funding agencies, it helps to find relationships between researchers and their work and the corresponding research data. Since the e!DAL infrastructure is generic and suitable for different kinds of research data, the ORCID system gives us an ideal solution to identify authors and improve the collected metadata for published datasets. Furthermore, the authors and their research data will get a better visibility, due to the connection between the ORCID infrastructure and infrastructure of the DataCite consortium, which is handling the DOIs.

To add the possibility for assigning an ORCID to every data creator or contributor in the e!DAL infrastructure, the original `PERSON` data type [17] in the e!DAL metadata schema was extended. e!DAL uses the REST API of the ORCID registry to provide the possibility to search for the ORCID of a given name. In addition, it can be validated if an entered ORCID belongs to the corresponding name to prevent an accidently linking with a wrong ORCID. All these API functions were integrated into the graphical user interface of the data submission tool for the PGP repository. Furthermore, the content pages of published and DOI linked datasets were improved to provide direct links to the ORCID profiles of the associated authors and contributors of the data.

JSON-LD & DC meta tags

Another method of making research data interoperable as well as machine-readable is to embed the describing metadata using *JavaScript Object Notation for Linked Data* (JSON-LD) format. This approach provides comprehensive possibilities to harvest and reuse research data. JSON-LD is a data serialization and exchange method and was developed to be easily embeddable into various systems for providing interoperable web services [38]. The dynamic HTML templates for the content pages of the embedded webserver of e!DAL, which provides the URLs for resolving the assigned DOIs, have been extended accordingly.

```
<script type ="application/ld+json">
{
   "@context":"http://schema.org",
   "@type":"Dataset",
   "@id":"https://doi.org/10.5447/IPK/2016/7",
   "name":"Raw images files from quantitative monitoring of...",
   "publisher":{
     "@type":"Organization",
     "name":"IPK Gatersleben"
   },
   "description":"This dataset contains 30426 raw image files...",
   "keywords":"high throughput plant phenotyping, growth protocol...",
   "inLanguage":"en",
   "author":[
      {
       "@type":"Person",
        "givenName":"Astrid",
        "familyName":"Junker",
        "address":"IPK Gatersleben"
      }
    ],
   "contributor":[
      {
       "@type":"Person",
       "givenName":"Thomas",
       "familyName":"Altmann",
       "address":"IPK Gatersleben"
      }
    ]
}
```

Listing 1. Reduced example of the JSON-LD data from the content page of a DOI assigned with e!DAL, which is stored in the PGP repository

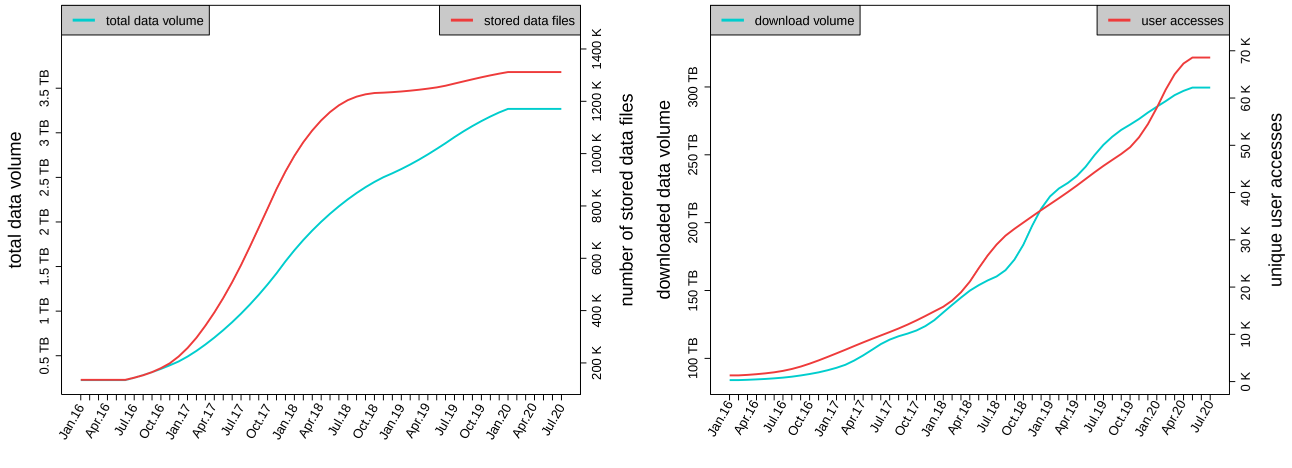

Figure 3. Access numbers and stock overview of the eDAL-PGP repository since 2016. The left diagram shows the development of the data volume and number of sored files, which were provided by the repository. The diagram on the right side the constantly increasing number of accesses and downloads of published datasets is shown.

Listing 1 show an example for the JSON-LD description of a dataset in the PGP repository. The attributes are based on the schema.org ontology, which is a well-established and community-driven vocabulary used to structure digital data on websites. It is used and harvested by several common search engines [39] and provides an interoperability between dataset from separated resources and platforms.

Another alternative to JSON-LD are so-called HTML meta tags. They are embedded in the `<head>` section of an HTML-document and also allow to harvest the metadata and describe connections between datasets from different infrastructures. As the metadata schema of the e!DAL infrastructure is already inspired by the DublinCore metadata schema [40] the embedded HTML templates for the content pages of published datasets were extended to provide the technical metadata of every object also as HTML meta tags (see Listing 2).

```
<meta name="DC.Title" content="Screening_of_wild_potato_genetic...">
<meta name="DC.Identifier" content="https://doi.org/10.5447/IPK/2019/1">
<meta name="DC.Publisher" content="e!DAL_-_Plant_Genomics_and_Phenomics...">
<meta name="DC.Language" content="en">
<meta name="DC.Description" content="This_data_set_contains_results_of...">
<meta name="DC.Rights" content="CC_BY-NC-SA_4.0">
<meta name="DC.Creator" content="Bachmann-Pfabe,_Silvia...">
<meta name="DC.Contributor" content="Dehmer,_Klaus...">
<meta name="DC.Subject" content="Phytophthora_infestans">
<meta name="DC.Subject" content="germplasm_collection">
```

Listing 2. Reduced example of the DublinCore Meta-Tags from the content page of a DOI assigned with e!DAL

Content Negotiation

Persistent DOIs provide a solution for long-term stable resolvability and referencing of all published datasets. In addition, for several reasons such as citing the datasets or harvesting the metadata, it is necessary to provide content negotiation to serve resources in different formats. Therefore the possibility to get different representations of the public datasets stored in an e!DAL infrastructure was implemented and can be used by several export functions, which were added on the corresponding content pages as shown in Figure 4. They provide the option to get textual representations, citation formats like BibTex or RIS and linked data formats like schema.org/JSON-LD and RDF for every dataset. Due to the fact that the DataCite service already provides a content negotiation feature, it was not necessary to implement a separate function for the embedded webserver of e!DAL. Instead, the HTTP handler uses the provided function for the different formats via a REST call and redirects the responses to the e!DAL infrastructure.

Elixir AAI

The e!DAL infrastructure provides a flexible and embedded security concept based on the Java Authentication and Authorisation Service (JAAS). To provide the research data management and publication capabilities to a wide range of users from universities, research institutes or further organisations, a new login module using the ELIXIR Authentication and Authorization Infrastructure (AAI) [41] was implemented. It was designed to provide a single sign-on service for authenticating researchers to services, which are a part of the ELIXIR portfolio. Doing so, it combines the huge amount of existing organisational identity providers from institutes that are associated with ELIXIR under one roof.

The new e!DAL login module follows the OAuth protocol [42] to authenticate users over the ELIXIR AAI and automatically receive their email address, which is necessary for the communication between the data submitting researcher and the reviewers of the embedded review process. Furthermore the email address is used as a kind of internal ID to authenticate the user within the e!DAL security system [17]. As the first use case, the new ELIXIR AAI based login was integrated into the PGP repository to open the infrastructure and the data

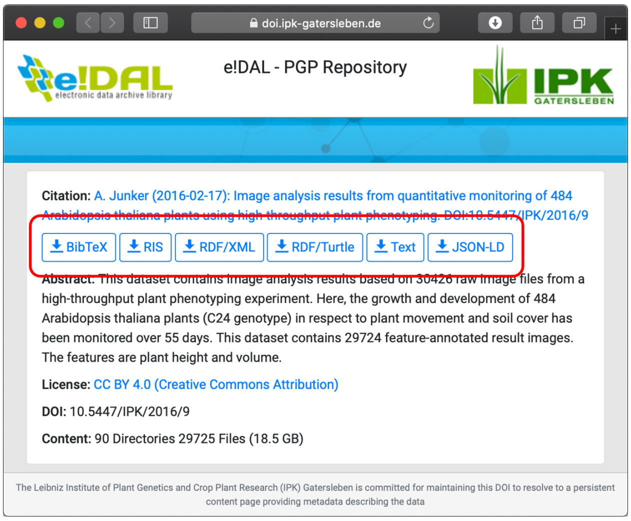

Figure 4. Screeshot of on example datasets in the PGP repository. The red colored rectangle mark the e!DAL embedded functions for the content negotiation.

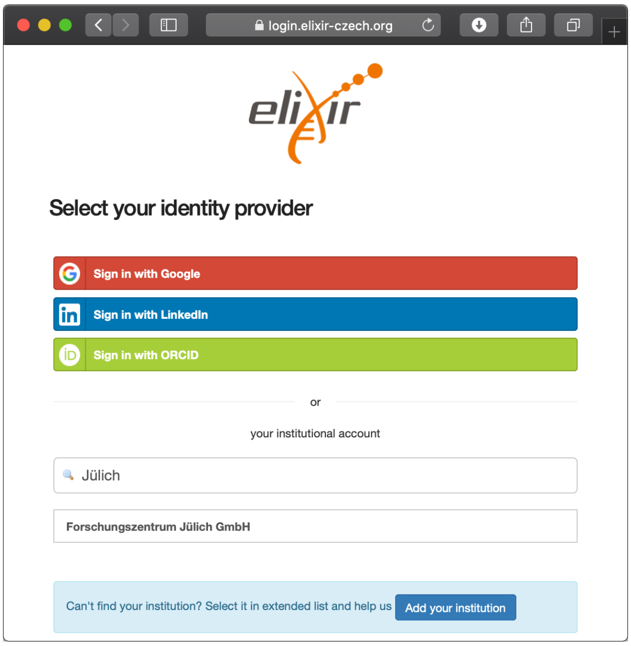

Figure 5. Integration of the ELIXIR AAI login dialog into the e!DAL infrastructure. The ELIXIR AAI provides beside a large collection of institutional identity providers, which can be easily found via the small search box, also several third party login opportunities based on Google, ORCID or LinkedIn. This provides the users a very flexible authentication infrastructure.

submission process for offering the service to a wide range of researchers without the need of creating a separate account. The ELIXIR AAI allows researchers to use their existing organisational accounts (see Figure 5), which lowers the barrier to use the infrastructure and to reach a larger group of data providers.

Furthermore, with the opportunity to use the ELIXIR AAI, the already low effort, which is necessary to establish further e!DAL installations, was reduced. Therefore at the end of 2018 a further e!DAL based repository at the Jülich Plant Phenotyping Center (JPPC) was established using the ELIXIR AAI login provider.

Amended frontend

The Apache Velocity template engine is used to render all HTML-based content of the e!DAL embedded webserver like the landing pages of published datasets and e-mail messages. This prevents the infrastructure from storing a massive amount of very similar websites and text drafts, which saves storage and provides a high performance for delivering content via the HTTP handler. All websites are provided dynamically on demand and created from only a few reusable templates.

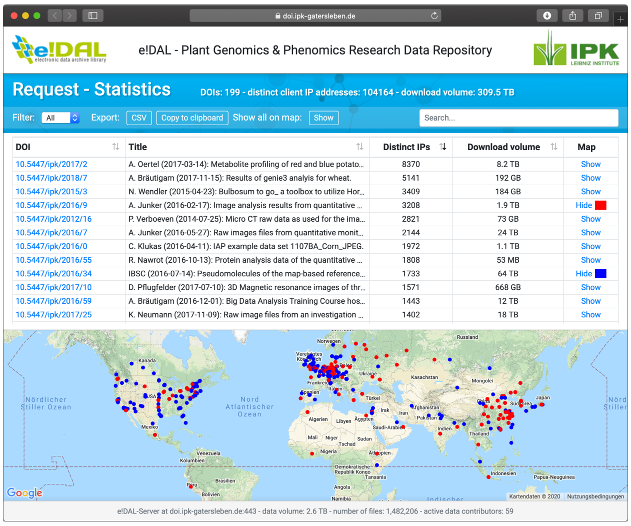

Figure 6. Example screenshot of the e!DAL embedded report page for the e!DAL-PGP repository showing the new layout and user interface. Several simple filter and search possibilities help to look for specific datasets. An integrated world map gives the user an idea on the dissemination of the data.

For the latest e!DAL version all content pages and the underlying templates were fully redesigned to provide a pleasing visual look and functional user experience. By using frontend frameworks and libraries like BootStrap and jQuery it is ensured that the user interface is responsive and working on both modern desktop browsers as well as on mobile devices. Figure 6 shows the new layout as an example screenshot of the embedded access statistic page of the PGP repository. Together with the new design for the frontend components of e!DAL, also the project website was renewed to provide comprehensive information for the user and for developers in a more concise manner.

Deployment and Usability

Since the last major release of the e!DAL infrastructure software a lot of optimizations and several new functionalities, which were described in the previous sections, have been implemented. Together with these improvements, changes in the general build and release process and in the usability have also been integrated. The most relevant of them are explained subsequently.

Gradle Multi-Build Project

After using the Maven build system Build System for several years for developing and releasing the e!DAL software components a change to the Gradle build tool was performed. Due to the constant increasing size of the project and the source code, because of new functionalities, several extensions and additional unit tests to guarantee a high software quality the build process using Maven takes quite a long time. This makes the regular release of stable versions very time-intensive. Furthermore, the build configuration became more complex and difficult to maintain. Gradle is strongly focused on a fast and specific build cycle. It supports multi-core systems to a high degree and allows e.g. the execution of several test suites in parallel. With the change to the build infrastructure, we also decided to redesign the entire project build hierarchy and created a multi-build project for the e!DAL infrastructure. It contains the main API components including the reference implementation as well as the components for the server-client architecture, which is directly based on this core implementation. This approach massively accelerates the build time, simplifies the maintenance and allows a more frequent deployment of new versions. The project is now available in a new BitBucket repository.

Nevertheless, the API is still released as an artifact in the central Maven Repository and can be integrated into other software projects using Maven or Gradle, as shown in Listing 3.

```
-------------------------- Maven - 'pom.xml' ------------------------------
<project>
  <dependencies>
    <dependency>
      <groupId>de.ipk-gatersleben</groupId>
      <artifactId>eDAL-MetaDataAPI | eDAL-MetaDataAPI-Server | eDAL-
            MetaDataAPI-Client</artifactId>
      <version>3.0.2</version>
    </dependency>
  <dependencies>
</project>
-------------------------- Gradle - 'build.gradle' ------------------------
repositories {
   mavenCentral()
}
dependencies {
   compile 'de.ipk-gatersleben:eDAL-MetaDataAPI:3.0.2'
   compile 'de.ipk-gatersleben:eDAL-MetaDataAPI-Server:3.0.2'
   compile 'de.ipk-gatersleben:eDAL-MetaDataAPI-Client:3.0.2'
}
```

Listing 3. Integration of the e!DAL components into the configuration of an Apache Maven or Gradle based project

OS specific executables

Due to the complete new development and release cycle by Oracle, the Java programming environment, which is the basis for the e!DAL infrastructure, changes a lot in recent years. In addition, the comprehensive redesign and reconstruction of the language itself, like the introduction of the new module concept or the removal of popular and formerly native APIs and frameworks like JavaFX or the Java Network Launching Protocol (JNLP), which was the basis for Java web start applications, were some very substantial changes. This strongly influences the e!DAL implementation, because they were also a significant part of the previous version. Unfortunately this impeded at some points the further development of e!DAL infrastructure, because a lot of the used frameworks and libraries needed several months to update their code to be compatible with the latest Java versions. With the new version 3.0.0 the e!DAL infrastructure is fully based on the Java Runtime Environment (JRE) 12. Therefore some comprehensive changes were necessary. In order to run e!DAL with the different existing runtimes, e.g. the official runtime from Oracle, but also the alternative and widely used OpenJDK, it was necessary to integrate the JavaFX library directly into the implementation. This increases the actual size of the API package, but it makes the infrastructure much more compatible and even more independent from the system preconditions than before.

The removal of the support for the popular and well known JNLP was also a high challenge, because the Java webstart tool was used to give the user an intuitive and platform-independent way to run the graphical data submission tool. Nevertheless this solution also provides some shortcomings like the need to provide an installed and compatible Java runtime. With the recently developed jpackage Java provides a powerful tool to pack self-contained applications along with

a suitable JRE. We used jpackage to create a full image of the e!DAL data submission tool together with a reduced JRE, which contains only the necessary java modules and provides separate executables for the most common operation system (Windows, Unix, MacOS). This provides a very convenient usability for data submitter and makes the infrastructure again more compatible and independent from the given system preconditions of the users.

Web-based submission application

In parallel to the update process due to the previously mentioned changes in the Java Runtime Environment and the development of the build process to create the self-executable applications for the submission dialog, a new web-based application was implemented to provide an alternative opportunity to upload research data to an e!DAL based infrastructure. The goal was the deployment of a user-friendly web application with the similar functionality of the corresponding desktop tool, but without the need to download the application as an executable or additional plugins. The Vaadin framework for Rich Internet Applications (RIA) was used for the implementation. Figure 7

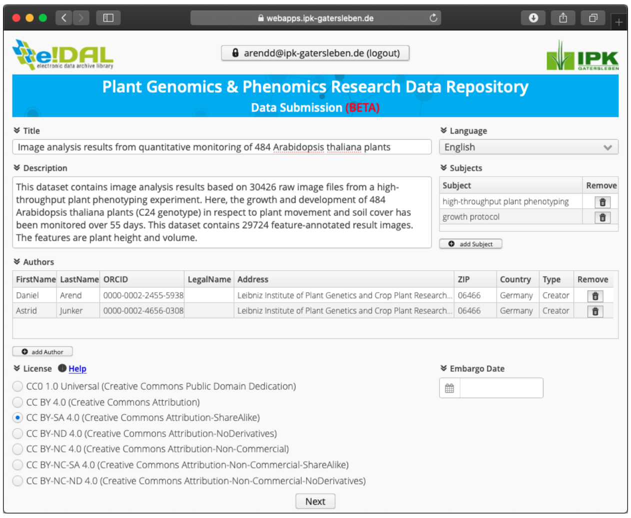

Figure 7. Example screenshot of the web-based submission application for the e!DAL-PGP repository. The form like user interface is very intuitive and similar to the desktop application. It helps the user to fill in the needed metadata by executing several checks to guarantee the completeness of the metadata.

shows a screenshot of the web application. By using several REST APIs, e.g. from the ORCID Registry or the ELIXIR AAI, a light-weight application could be created providing the same functionality as the full desktop client. Furthermore,users now have the possibility to submit research data also from mobile devices or other browser compatible devices. The only small shortcoming of the data submission via the web application is currently that not all browsers support the upload of comprehensive file folders. The latter is only possible if a recent version of Google Chrome or Mozilla Firefox is used. Other web browsers only allow the upload of single files.

Results

In this article the basic overall 'on-premise' data management and publication concept of the e!DAL infrastructure as well as several new features and technical developments were presented. As a result, e!DAL matured to a comprehensive and FAIR-compliant infrastructure, while always keeping the focus on simple and flexible setup and integration into existing infrastructures and into the daily research process. With the described 'bring the infrastructure to the data' approach, it differs fundamentally from generic publication platforms like figshare or DRYAD, which can produce, depending on the needed storage, considerable financial costs and time costs for transferring the data. e!DAL allows the usage of available in-house storage capacities, without the need of complex requirements and technical infrastructures or comprehensive adaptations. All functionalities are already included and the provided reference implementation contains required components, such as a database or a webserver. This is a crucial advantage in comparison to other similar software infrastructures, like DataVerse or CKAN, and lowers the barrier to establish a publication infrastructure even for small-size research institutions with limited possibilities and know-how.

Thereby the FAIR compliance can be fulfilled by several e!DAL functions and components:

- **Findable:**
  By providing embedded and machine-readable metadata based on standardized established formats, the e!DAL published datasets can be easily found using common search engines like Google or the DataCite Metadata Search. Due to the widely established and used DOIs, the DataCite consortium is also involved in several projects and interacts with different systems like ORCID, CrossRef or Scholix. This further improves the findability of e!DAL datasets.
- **Accessible:**
  e!DAL fully support the usage of DOIs as persistent identifiers to guarantee a long-term stable availability of published datasets. The DataCite resolver for the DOIs allows simple access to the data and reference datasets, e.g. in a research article or as part of data publication. If the storage location of the underlying data is changing, the corresponding DOI remains stable and allows the uninterrupted access to the data by updating the resource path. At that point the embedded web server of e!DAL takes care that every published DOI is accessible via a comprehensive content page. It provides the opportunity to navigate through the dataset and download certain files, and furthermore the access of the metadata and a direct linkage to the ORCID registry.
- **Interoperable:**
  To provide interoperable datasets and to allow the aggregation of information about the relationship of datasets from different sources, the e!DAL infrastructure supplies embedded metadata on the content pages of every data object. They are stored using standardized formats and vocabularies like JSON/LD or rather schema.org.
- **Reusable:**
  By collecting a standardized set of mainly technical metadata e!DAL guarantees a long-term readability and usability of all published datasets. The schema is inspired by the DublinCore metadata format and meets community established standards. Furthermore, a clear and easy license handling allows to assign a suitable license, which defines by whom and how the data can be used. They are available both on the content page of every data object as well as embedded in the HTML sources.

e!DAL Usage

Established in 2016, the PGP repository is the first productive repository based on the e!DAL infrastructure and a part of the service portfolio of the GCBN unit (German Crop BioGreenformatics Network) [25] of de.NBI (German Network for Bioinformatics Infrastructure) [43], which is the head of ELIXIR Germany. After more than three years of productive usage, the PGP repository currently shares comprehensive, plant-related research datasets containing mainly genomic and phenomic

information, but also metabolic datasets or software components and pipelines. Most of the datasets are part of a corresponding research paper and allow authors from IPK, but also from other institutes, to improve their manuscripts by enriching them with the underlying research data in a FAIR compliant way. The overall download volume and large number of distinct user accesses show the high visibility of the provided data sets and the interest of the research community for this kind of research data.

The integration of the ELIXIR AAI into the login mechanism of the PGP Repository is a prime example that shows how established platforms can benefit from the ELIXIR network. The provided services contribute to the increase of the visibility, to overcome the obstacles for the use of available infrastructures and to support FAIR compliant access to research data. The support of the ELIXIR single sign-on service enables collaborators to use the PGP repository as a service to publish their research data. Furthermore, the ELIXIR AAI login is fully integrated into the e!DAL infrastructure software, which allows to set up further FAIR in-house repository instances, following the presented I2D approach. Doing so in June 2018 a second repository based on the developed e!DAL infrastructure was established at the *Forschungszentrum Jülich*. Due to the auto-configuring installation it was possible to run the system and provide the submission and review workflow with only a little effort in time. The integrated ELIXIR AAI login allows researchers from Jülich to use their existing institutional accounts. The complete infrastructure is hosted and maintained by the *Jülich Plant Phenotyping Center (JPPC)*. The process of establishing further e!DAL-based repositories at the Julius-Kühn Institute and the Helmholtz Centre München are currently underway.

Outlook

In this work, we showed the newly designed I2D concept for FAIR compliant data publication by using in-house storage infrastructures and new features of the e!DAL platform. After several years of operating a productive instance of this infrastructure as the basis for the PGP repository, we recorded high numbers of accesses and downloads. Although researchers have more and more possibilities to share their research data with the community, the incentive to do so is still not high enough for some researchers [44]. In contrast to the common peer-reviewed publication in journals, it is not so easy to measure the impact of research data itself, because the concept of data citation is still not a common practice [45], but it becomes more and more important and accepted [46]. It's not only a cultural problem, but also a technical challenge and therefore an issue of practicability[47]. One of the first metrics to count data citations was the commercial Data Citation Index. But in the meanwhile some free and community initiated projects like Make Data Count have been developed. Furthermore, popular journals are starting to demand that authors put their research data as data citations in their common reference list [48]. This facilitates to measure its impact through a citation index and improves the visibility to readers which in turn increases the general acceptance of research data as valuable scientific assets. In future we will investigate several approaches for counting data citations and getting more credit for publishing research data. We plan to integrate a generic and open-source solution into the e!DAL infrastructure to show users comprehensive information how their data is reused and referenced.

The ORCID provide a widely accepted and used solution to unambiguously identify researchers. The integration within the e!DAL infrastructure is very intuitive and facilitates handling of multiple ORCIDs for comprehensive lists of authors. Besides the identification of persons, it can be also quite challenging to handle the diverse affiliations of research institutes, universities or companies with a focus on different scientific topics. Some authors have multiple affiliations, from time to time organizations may be renamed, the official addressee may change due to infrastructural developments or it may happen that an institute will be closed. The Research Organization Registry (ROR) provides an open and sustainable approach, which is led by the community and supported by popular organizations like DataCite or Dryad. The concept of the ROR identifiers is very similar to the ORCIDs and allows to uniquely identify all kinds of research organizations. Therefore, one of the next functional improvements for the e!DAL infrastructure will be the integration using the provided ROR API. This will cause some changes in the basic data structure, which however will result in a much easier and FAIRer way to handle author affiliations [49].

Availability of source code and requirements

- Project name: e!DAL (electronic Data Archive Library)
- Current version: 3.0.2
- Project homepage: `https://edal.ipk-gatersleben.de`
- Source Code Repository: `https://bitbucket.org/ipk_bit_team/electronicdataarchivelibrary`
- Operating system(s): platform independent
- Programming language: JVM based (Java 12+)
- JavaDoc: `https://edal.ipk-gatersleben.de/javadoc`
- Artifact repository: Maven Central (`https://mvnrepository.com/artifact/de.ipk-gatersleben`)
- License: GNU General Public License (GPL) Version 3 (`https://www.gnu.org/licenses/gpl-3.0.html`)
- BioTools: `https://bio.tools/edal`
- RRID: electronic Data Archive Library, RRID:SCR_019017

The e!DAL project website provide comprehensive information, which are relevant for users as well as developers. Beside a full Java documentation, further presentations, videos and several code and usage examples are presented. We changed the licence model of e!DAL to GNU General Public License (GPL) Version 3. This aim at maximising the spread of the e!DAL infrastructure in scientific community to foster FAIR principles of in-house stored data and to enable the incorporation of e!DAL into 3rd party software as well.

Declarations

List of abbreviations

AAI: Authentication and Authorization Infrastructure;
API: Application programming interface;
de.NBI: German Network for Bioinformatics Infrastructure;
DOI: Digital Object Identifier;
DPPN: German Plant Phenotyping Network;
FAIR: Findable, Accessible, Interoperable, Reusable;
GCBN: German Crop BioGreenformatics Network;
I2D: Infrastructure-to-the-Data (I2D);
JNLP: Java Network Launching Protocol;
JPPC: Jülich Plant Phenotyping Center;
JRE: Java Runtime Environment;
JSON-LD: JavaScript Object Notation for Linked Data;
ORCID: Open Researcher and Contributor ID;
PGP: Plant Genomics and Phenomics Research Data Repository;
PGR: Plant Genetic Resources;
RIA: Rich Internet Application;
RMI: Remote Method Invocation;

Competing Interests

The authors declare no conflict of interest.

Funding

This work was supported by the German Federal Ministry of Education and Research (BMBF) in frame of the the projects German-Plant-Phenotyping Network - DPPN (FKZ 031A053), Modernste Virtualitäts- und erweiterte Realitäts-Verfahren für den Zyklus von Samen zu Samen - AVATARS (FKZ 031B0770A) and German Network for Bioinformatics Infrastructure - de.NBI (FKZ 031A536A).

Author's Contributions

Conceptualization: DA,ML
Software: DA,PK
Investigation: DA
Supervision: ML
Writing original draft: DA,ML
Writing review & editing: All
Funding acquisition: US,AJ,ML

Acknowledgements

We want to thank Thomas Münch, Jens Bauernfeind and Heiko Miehe for the technical support. Furthermore we are very thankful to Benjamin Bruns, Robin Kluth and Michael Seidel for fruitful discussions, testing the infrastructure and formulating feature requests. Last but not least we thank all users and data producers for their valuable feedback.

References

1. Martone ME. FORCE11: Building the Future for Research Communications and e-Scholarship. BioScience 2015;65(7):635. https://doi.org/10.1093/biosci/biv095.
2. Wilkinson MD, Dumontier M, Aalbersberg IJ, Appleton G, Axton M, Baak A, et al. The FAIR Guiding Principles for scientific data management and stewardship. Scientific data 2016;3. https://doi.org/10.1038/sdata.2016.18.
3. European Commission – Directorate-General for Research and Innovation. Guidelines on FAIR Data Management in Horizon 2020; 2016, http://ec.europa.eu/research/participants/data/ref/h2020/grants_manual/hi/oa_pilot/h2020-hi-oa-data-mgt_en.pdf.
4. Mons B, Neylon C, Velterop J, Dumontier M, da Silva Santos LOB, Wilkinson MD. Cloudy, increasingly FAIR; revisiting the FAIR Data guiding principles for the European Open Science Cloud. Information Services & Use 2017;37(1):49–56. https://doi.org/10.3233/ISU-170824.
5. Burgelman JC, Pascu C, Szkuta K, Schomberg RV, Karalopoulos A, Repanas K, et al. Open Science, Open Data, and Open Scholarship: European Policies to Make Science Fit for the Twenty-First Century. Frontiers in Big Data 2019 dec;2. https://doi.org/10.3389/fdata.2019.00043.
6. figshare;. https://figshare.com/.
7. Zenodo;. https://zenodo.org/.
8. Dryad;. https://datadryad.org/.
9. Wolstencroft K, Krebs O, Snoep JL, Stanford NJ, Bacall F, Golebiewski M, et al. FAIRDOMHub: a repository and collaboration environment for sharing systems biology research. Nucleic Acids Research 2016 nov;45(D1):D404–D407. https://doi.org/10.1093/nar/gkw1032.
10. Durinx C, McEntyre J, Appel R, Apweiler R, Barlow M, Blomberg N, et al. Identifying ELIXIR Core Data Resources. F1000Research 2017;5(2422). https://doi.org/10.12688/f1000research.9656.2.
11. Coordinators NR. Database resources of the national center for biotechnology information. Nucleic acids research 2018;46(Database issue):D8. https://doi.org/10.1093/nar/gkv1290.
12. Leinonen R, Sugawara H, Shumway M, Collaboration INSD. The sequence read archive. Nucleic acids research 2010;39:D19–D21. https://doi.org/10.1093/nar/gkq1019.
13. Submission System of the European Nucleotide Archive;. https://www.ebi.ac.uk/ena/submit.
14. Submission System of the European Variation Archive;. https://www.ebi.ac.uk/eva/?Submit-Data.
15. Papoutsoglou EA, Faria D, Arend D, Arnaud E, Athanasiadis IN, Chaves I, et al. Enabling reusability of plant phenomic datasets with MIAPPE 1.1. New Phytologist 2020 mar;p.nph.16544. https://doi.org/10.1111/nph.16544.
16. ISA-Tab-for-plant-phenotyping;. https://github.com/MIAPPE/ISA-Tab-for-plant-phenotyping.
17. Arend D, Lange M, Chen J, Colmsee C, Flemming S, Hecht D, et al. e!DAL – a framework to store, share and publish research data. BMC Bioinformatics 2014 Jun;15(1):214. https://doi.org/10.1186/1471-2105-15-214.
18. Brase J. DataCite-A global registration agency for research data. In: 2009 Fourth International Conference on Cooperation and Promotion of Information Resources in Science and Technology IEEE; 2009. p. 257–261. https://doi.org/10.2139/ssrn.1639998.
19. Arend D, Junker A, Scholz U, Schüler D, Wylie J, Lange M. PGP repository: a plant phenomics and genomics data publication infrastructure. Database 2016;2016:baw033. https://doi.org/10.1093/database/baw033.
20. Halewood M, Chiurugwi T, Sackville Hamilton R, Kurtz B, Marden E, Welch E, et al. Plant genetic resources for food and agriculture: opportunities and challenges emerging from the science and information technology revolution. New Phytologist 2018;217(4):1407–1419. https://doi.org/10.1111/nph.14993.
21. Ferranti P. Preservation of Food Raw Materials. In: Reference Module in Food Science Elsevier; 2016.https://doi.org/10.1016/b978-0-08-100596-5.03445-4.
22. Milner SG, Jost M, Taketa S, Mazón ER, Himmelbach A, Oppermann M, et al. Genebank genomics highlights the diversity of a global barley collection. Nature genetics 2019;51(2):319. https://doi.org/10.1038/s41588-018-0266-x.
23. Rosenqvist E, Großkinsky DK, Ottosen CO, van de Zedde R. The Phenotyping Dilemma—The Challenges of a Diversified Phenotyping Community. Frontiers in Plant Science 2019;10:163. https://doi.org/10.3389/fpls.2019.00163.
24. Oppermann M, Weise S, Dittmann C, Knüpffer H. GBIS: the information system of the German Genebank. Database 2015;2015. https://doi.org/10.1093/database/bav021.
25. Schmutzer T, Bolger ME, Rudd S, Chen J, Gundlach H, Arend D, et al. Bioinformatics in the plant genomic and phenomic domain: The German contribution to resources, services and perspectives. Journal of Biotechnology 2017;261:37 – 45. https://doi.org/10.1016/j.jbiotec.2017.07.006.
26. Leonelli S, Davey RP, Arnaud E, Parry G, Bastow R. Data management and best practice for plant science. Nature Plants 2017 jun;3(6). https://doi.org/10.1038/nplants.2017.86.
27. Leinonen R, Akhtar R, Birney E, Bower L, Cerdeno-Tárraga

A, Cheng Y, et al. The European Nucleotide Archive. Nucleic Acids Research 2011;39:D28–D31. https://doi.org/10.1093/nar/gkq967.
28. Apweiler R, Bairoch A, Wu CH, Barker WC, Boeckmann B, Ferro S, et al. UniProt: the Universal Protein knowledgebase. Nucleic Acids Research 2004;32:D115–D119. https://doi.org/10.1093/nar/gkh131.
29. Perez-Riverol Y, Csordas A, Bai J, Bernal-Llinares M, Hewapathirana S, Kundu DJ, et al. The PRIDE database and related tools and resources in 2019: improving support for quantification data. Nucleic Acids Research 2018 nov;47(D1):D442–D450. https://doi.org/10.1093/nar/gky1106.
30. Le Novère N, Bornstein B, Broicher A, Courtot M, Donizelli M, Dharuri H, et al. BioModels Database: a free, centralized database of curated, published, quantitative kinetic models of biochemical and cellular systems. Nucleic Acids Research 2006;34(suppl 1):D689–D691. https://doi.org/10.1093/nar/gkj092.
31. Crosswell LC, Thornton JM. ELIXIR: a distributed infrastructure for European biological data. Trends in Biotechnology 2012 may;30(5):241–242. https://doi.org/10.1016/j.tibtech.2012.02.002.
32. Schomburg I, Jeske L, Ulbrich M, Placzek S, Chang A, Schomburg D. The BRENDA enzyme information system – From a database to an expert system. Journal of Biotechnology 2017;261:194 – 206. https://doi.org/10.1016/j.jbiotec.2017.04.020.
33. Quast C, Pruesse E, Yilmaz P, Gerken J, Schweer T, Yarza P, et al. The SILVA ribosomal RNA gene database project: improved data processing and web-based tools. Nucleic Acids Research 2013;41(D1):D590–D596. https://doi.org/10.1093/nar/gks1219.
34. Chavan V, Penev L. The data paper: a mechanism to incentivize data publishing in biodiversity science. BMC Bioinformatics 2011 Dec;12(15):S2. https://doi.org/10.1186/1471-2105-12-S15-S2.
35. Fenner M, Crosas M, Grethe JS, Kennedy D, Hermjakob H, Rocca-Serra P, et al. A data citation roadmap for scholarly data repositories. Scientific Data 2019;6(1):28. https://doi.org/10.1038/s41597-019-0031-8.
36. Haak LL, Fenner M, Paglione L, Pentz E, Ratner H. ORCID: a system to uniquely identify researchers. Learned Publishing 2012;25(4):259–264. https://doi.org/10.1087/20120404.
37. Dreyer B, Hagemann-Wilholt S, Vierkant P, Strecker D, Glagla-Dietz S, Summann F, et al. Die Rolle der ORCID iD in der Wissenschaftskommunikation: Der Beitrag des ORCID-Deutschland-Konsortiums und das ORCID-DE-Projekt. ABI Technik 2019 jul;39(2):112–121. https://doi.org/10.1515/abitech-2019-2004.
38. Consortium WWW, et al. JSON-LD 1.0: a JSON-based serialization for linked data 2014;http://hdl.handle.net/10421/7478.
39. Guha RV, Brickley D, Macbeth S. Schema.org: evolution of structured data on the web. Communications of the ACM 2016 jan;59(2):44–51. https://doi.org/10.1145/2844544.
40. Weibel S. The Dublin Core: A Simple Content Description Model for Electronic Resources. Bulletin of the American Society for Information Science and Technology 1997;24(1):9–11. https://doi.org/10.1002/bult.70.
41. Linden M, Prochazka M, Lappalainen I, Bucik D, Vyskocil P, Kuba M, et al. Common ELIXIR Service for Researcher Authentication and Authorisation [version 1; peer review: 3 approved, 1 approved with reservations]. F1000Research 2018;7(1199). https://doi.org/10.12688/f1000research.15161.1.
42. Hardt D; Internet Engineering Task Force. The OAuth 2.0 Authorization Framework (RFC 6749) 2012;https://doi.org/10.17487/RFC6749.
43. Tauch A, Al-Dilaimi A. Bioinformatics in Germany: toward a national-level infrastructure. Briefings in Bioinformatics 2017 04;20(2):370–374. https://doi.org/10.1093/bib/bbx040.
44. Cousijn H, Feeney P, Lowenberg D, Presani E, Simons N. Bringing Citations and Usage Metrics Together to Make Data Count. Data Science Journal 2019;18(1). https://doi.org/10.5334/dsj-2019-009.
45. Konkiel S. Tracking citations and altmetrics for research data: Challenges and opportunities. Bulletin of the American Society for Information Science and Technology 2013;39(6):27–32. https://doi.org/10.1002/bult.2013.1720390610.
46. Tenopir C, Dalton ED, Allard S, Frame M, Pjesivac I, Birch B, et al. Changes in Data Sharing and Data Reuse Practices and Perceptions among Scientists Worldwide. PLOS ONE 2015 aug;10(8):e0134826. https://doi.org/10.1371/journal.pone.0134826.
47. Parsons MA, Duerr RE, Jones MB. The History and Future of Data Citation in Practice. Data Science Journal 2019;18. https://doi.org/10.5334/dsj-2019-052.
48. Data citation needed. Scientific Data 2019;6(1):27. https://doi.org/10.1038/s41597-019-0026-5.
49. Hahnel M, Valen D. How to (Easily) Extend the FAIRness of Existing Repositories. Data Intelligence 2019 nov;p. 192–198. https://doi.org/10.1162/dint_a_00041.

Click here to access/download;Manuscript;gigascience.pdf

Your PDF file "gigascience.pdf" cannot be opened and processed. Please see the common list of problems, and suggested resolutions below.

Reason:

Other Common Problems When Creating a PDF from a PDF file

You will need to convert your PDF file to another format or fix the current PDF file, then re-submit it.
