# Supplementary material for: The on-premise data sharing infrastructure e!DAL: Foster FAIR data for faster data acquisition
Source: Gigascience. 2020 Oct 22;9(10):giaa107. doi: 10.1093/gigascience/giaa107 (PMC7580168; doi:10.1093/gigascience/giaa107)
Supplement: giaa107_Response_to_Reviewer_Comments_Original_Submission [file giaa107_response_to_reviewer_comments_original_submission.pdf]

Author's Response To Reviewer Comments

Close

Comments and responses to reviewer comments

Reviewer 1:

Summary

The manuscript "The on-premise data sharing infrastructure e!DAL: Foster FAIR data for faster data acquisition" by Arend and colleagues provides an overview of updates and enhancements to the e!DAL on-premise data-sharing platform. The authors address common issues with scientific data sharing using centralized repositories: 1) data (potentially a large quantity) needs to be transferred from the owner to a repository; 2) data often need to repackaged in various ways to fit the expectations of the repository; 3) dataset metadata needs to potentially be tailored to each repository; 4) costs associated with central repositories (direct or indirect, e.g. training); and 5) lack of central repositories for some data types (e.g. imaging and phenotype data). To address these issues, e!DAL provides the infrastructure to achieve data sharing using FAIR principles using on-premise data infrastructure. e!DAL provides a data publication layer on top of a data repository that interfaces with existing publication and search engine infrastructure to make shared datasets FAIR.

Strengths
----
* The manuscript is well-written and succinctly describes the rationale for and improvements of e!DAL.
* The figures effectively summarize the major features of e!DAL and the results of its usage.

Weaknesses
----
* None

We want to thank the reviewer for his positive feedback and effort to review our manuscript.

Suggestions for improvement

My understanding is that GPL licenses require an exact copy of the license be distributed with the software: https://www.gnu.org/licenses/gpl-faq.html#WhyMustIInclude. The current license file(s) in e!DAL only states that GPLv3 applies but there does not seem to be a copy of GPLv3 itself.

Thank you for the hint, we added a full version of the license text to the license file in the Bitbucket repository of e!DAL.
https://bitbucket.org/ipk_bit_team/electronicdataarchivelibrary/src/master/LICENCE.md

The manuscript discusses that "By collecting a standardized set of mainly technical metadata e!DAL guarantees a long-term readability and usability of all published datasets." This is important for making datasets findable, but there is an additional layer often required to use data, which is documentation of what the data is, how it was collected, how it is structured, etc. While not in the scope of the current work, could the authors potentially discuss in the outlook section whether they see this type of metadata or documentation being a component of e!DAL? In particular, I am thinking of metadata ontologies like MIAPPE, MIxS, etc.

You are right, of course semantic metadata is an important point. But the e!DAL concept to expose even “grey” and semi-structured research data is applied to the metadata too. Here we differentiate between generic, technical metadata, which are stored within e!DAL and specific semantic metadata. We are aware of the trade-off to make as much research data as possible FAIR and expose high quality semantic metadata. We think it is beneficial to expose datasets even without mandatory semantic metadata. Because it is still a resource challenge for scientists to annotate research data accordingly.

Due to the strong heterogeneity and diversity of research data and semantic metadata schema as well technical challenges, this has to be accompanied by institutional policies and Data Stewards. However, until a general cultural change and its wide implementation in the research landscape, we aim minimally at exposing research data even with technical metadata only. The major goal of the development was providing a generic infrastructure that could be set-up and integrated easily. Supporting specific semantic metadata schemas like MIAPPE would need massive effort and institute specific adaptation for every instance. Therefore we think it is the task for the hosting institute or rather the specific reviewers of ever instance to take care that every dataset that they accept is in the scope of the certain instance and provide suitable semantical metadata, while e!DAL takes care that the technical metadata, which are needed to guarantee long-term readability and which are necessary to assign a DOI, are provided. For example, the mentioned PGP repository, which is hosted at the IPK Gatersleben focuses among others on plant phenotypic data. Therefore our reviewers carefully check if every submitted dataset providing phenotypic data contains a MIAPPE compliant metadata description. Other e!DAL based repositories may have a focus on
different data domains and therefore evaluate every submission
differently.

Reviewer 2:

This manuscript provides a clear update on the e!DAL software package for the operation of local data repository infrastructure. Following the "Infrastructure to data (I2D)" model, the tool is available for reuse and is currently operational in two institutions (e.g. operating the "Plant Genomics and Phenomics Data Repository (PGP)" at IPK-Gatersleben) with a further two currently installing it. The manuscript provides details on some appropriate updates and additions that include enhanced data ingress performance, addition of the ORCID identifier system for contributor authentication, the ELIXIR AAI, JSON-LD presentation format, use of Gradle build/deployment infrastructure and a choice of two data upload/submission routes - full application and web application.

We want to thank the reviewer for his positive feedback and effort to review our manuscript

e!DAL and its implementations (especially PGP, which could be considered the "reference" implementation), lie within the ELIXIR ecosystem. As the authors note, e!DAL occupies a niche in this ecosystem that is as yet unfilled for many of the data types associated with plant phenomics. However, it also has in scope data of types that can be handled by other elements of the ecosystem, such as the ELIXIR Core Data Resources. These include, for example, genomics databases (see a data set of relevance for this, for example, at https://doi.ipk-gatersleben.de/DOI/1c5dc9c8-0b38-4b2b-93d3-993272532cb1/711ad917-d85b-4e08-b883-8af94ae215b0/2). I recommend that the authors address this issue in the system and the manuscript: how does the system ensure that incoming data sets that include data types appropriate for deposition elsewhere in ELIXIR are appropriately routed and linked from the system?

Yes you are right, the mentioned dataset would also fit into established genomic databases. As we also mention in the following question, the reviewers are mainly responsible to take care about the data quality and that the dataset fits into the scope of the repository. In the case of the mentioned dataset the reason for its deposition into the e!DAL-PGP repository was due to the less strict recommendation policy in Nature for the publication of miRNA loci and the necessity of an ad-hoc publication of this data set to meet the submission deadlines for the paper (https://dx.doi.org/10.1038/nature22043). However,

we are aware that choosing the right public repository is an important issue. We consider this in the review process and always try to recommend the most appropriate repository for data submission.

While promoting FAIR principles, it is not clear from the manuscript how e!DAL supports compliance for data sets with community data standards. I would have expected reference in the text, for example, to ELIXIR-related data standards for plant sciences such as MIAPPE and the Breeding API (https://elixir-europe.org/communities/plant-sciences).

Of course you are right it is important to support and push established community standards, but this is very difficult due to the strong heterogeneity of research data and not objective of the generic e!DAL software, because it is data domain agnostic. This is rather a task of the data submitters to take care that he uses established standards and metadata schema to provide FAIR research data. Nevertheless, we agree this should be the task of the reviewers of the specific e!DAL based repositories. However, we see a gap between FAIR culture and its implementation in projects by dedicated resources and monitored processes whereas every dataset needs to be checked in respect of data and metadata quality. As argued before, it is a matter of policies and resources at the site of data producer and reviewer, too.

e!DAL takes the I2D model in which distinct repositories operate at institutional level and are connected through the DOI system of identifiers. The authors lay out some but not all of the features of such a model. While they correctly declare that in the event that an e!DAL repository is removed from service, metadata relating to its content will remain in the DataCite system, this is far from optimal as the data themselves will have been lost. In the I2D model, what mechanisms exist, or could be put in place, to protect against such loss?

That is a very good analysis of the described I2D model. There is no out-of-the-box solution within the e!DAL infrastructure software, but due to the I2D concept is the task of the hosting institute to protect against data loss by protecting the hardware components on which the e!DAL instance is running. For example the mentioned e!DAL-PGP repository, which is hosted at the IPK Gatersleben, is using a powerful HSM (hierarchical storage management) infrastructure, which backups the stored datasets and protects them in case of hardware failures or other issues. In addition, the other instances running at the research centre Jülich or at the JKI are using similar backup solutions based on their local, institutional hardware infrastructure. Maybe in the future it is worth thinking about an embedded support within the e!DAL software like providing an option to set-up the infrastructure on distributed systems to help the installing institute to protect against data loss, but this could be quite challenging and would also increase the needed effort to set-up repositories based on the e!DAL.

Reviewer 3:

This paper describe a sustainable data repository solution that provides an intermediate between the most important international data repositories and non-sustainable project databases. It provides an

alternative to Research institutions dataverses or dspace with the promise of reduced deployment costs. This paper therefore discuss a very important question.

The alternate software solutions are fairly presented and the advantages of e!DAL are correctly discussed.

The paper is well written and organized. Some modifications are proposed below.

The software and data repositories are publicly accessible and the software is under GPLv3 open source license. The source code repository is missing a LICENSE file though.

The technical details are clearly introduced and discussed with sufficient information. The reuse of the code is well documented but I haven't tested it.

Therefore the proposition is to request Minor revisions before publishing this quality article.

We want to thank the reviewer for his very detailed feedback and effort to review our manuscript.

Remarks:

Some references lack DOI Nature genetics, New Phytologists, …)

Thank you for the hint, we checked all references again and added missing DOIs

DOI minting is not discussed, it could be a plus as getting DOI as a cost for an organization.

We understand the question in two aspects. First situation is that an external user submits his data to an existing e!DAL repository, like the PGP repository at the IPK. Then of course he can get his DOI for free, because the IPK as a hosting institute is registered as a data centre in the DataCite consortium and pays for DOI minting. On the other side if another institution decided to use e!DAL to set-up his own instance then they have to take care to become a DataCite member to get an own account for their repository to mint DOIs. But anyway our experience is that for many institutes in the past it was not a major challenge to get a DataCite account or to pay for the DOI minting, but it was missing a suitable infrastructure software to organize their datasets and to assign a DOI. And with e!DAL they have an easy and generic solution for this problem.

A GPLv3 license file should be added in the eDALE code repository.

Thank you for the hint, we added a full version of the license text to the license file in the Bitbucket repository of e!DAL.
https://bitbucket.org/ipk_bit_team/electronicdataarchivelibrary/src/master/LICENCE.md

-- Abstract-- :
« the storage of which is not covered by established core databases " : There are established databases, some are Elixir Core for Genomic and others only established for Phenomics.

Yes, you are right this was poorly formulated. We modified this section in the abstract to make this point as well as the following one more clear.

"Due to its high volume and strong heterogeneity, resulting in missing infrastructures" : this sentence should be clarified.

See previous answer.

"ELIXIR AAI » : the purpose of this service isn't obvious for non Elixir reader.

Yes that is absolutely true, due to the word limit in the abstract we have to avoid to go into detail here, but we added the full form of AAI (‘Authentication and Authorization Infrastructure’), because this should give an idea of the purpose even for non ELIXIR readers.

"as means tolower « : typo

We corrected the typo.

--Introduction--
"general purpose data repositories, e.g. figshare [6], Zenodo [7] and Dryad [8] " : FAIRDOM is another repository that might be worth mentioning.

Yes, thanks for the idea, we added FAIRDOM.

"(iii) institutional ": research institute might better reflect the authors intention

Yes that is correct, we reformulated that point.

", e.g. the EBI and NCBI core data resources, Bioinformaticians are charged and trained ": the end of this sentence should be clarified

We changed the sentence a little bit to make clear that bioinformaticians are often necessary due to the diverse and specific submission requirement for some repositories.

"or the preparation of ISA-TAB compatible data submission for plant phenotyping data [14, 15]. ": MIAPPE paper: this is the first one, two others have been published since 2015. The latest should be the most accurate.

That is correct, we replaced the reference with the latest article: https://doi.org/10.1111/nph.16544

"Alternatively, project-related or institutional data repositories could be set up. " Can Research institution repositories and project databases be really placed at the same level ?

Yes, you are right this is poorly formulated. We changed the sentence.

"This finally enables the assignment of DOIs with a minimal set of metadata to in-house stored data and its approved FAIR refer- encing by journals or data lookup services. ": it would be worth describing a little be more the metadata (Dublin core minimal dataset or a more extensive list of minimal information about… MIA*)

The metadata are technical and administrative metadata and based on the DublinCore. We added a short phrase to clarify this. Details are already described in the first e!DAL publication, which is also cited in the same section (http://dx.doi.org/10.1186/1471-2105-15-214). Therefore we have not gone into detail here.

"Approximately seven million crop accessions « : the term accession should be describe a little bit more for new readers (eg PGR accession)

Thanks for the hint, we added an additional sentence and reference, as well as reformulated the phrase.

"that do not fit into classical databases due to their volume " : What are precisely those classical databases ? It would be good to refer to the three categories of the first paragraph.

We reformulated the sentence to clarify this and refer to the previous mentioned types in the first paragraph.

"This experience and the adoption as a service in the European life-sciences Infrastructure for biological Information ELIXIR [23] " : the words "adoption as a service" would need clarification or rephrasing.

Yes, this is correct, “adoption as a service” seems to be a quite strong wording. We rephrase the sentence and also add a link to the list of ELIXIR services.

-- Related Work --

"Most of them evolved over many years and they are widely accepted by the research community [24] " the reference 24 is a bit old (2010) if possible a newer one would be a plus.

Thanks for the hint, we added a newer publication: https://doi.org/10.1038/nplants.2017.86

"Usually also research journals and other publishers request data sharing using these established domain- specific databases. " They require the use of sustainable repositories which can be found using means described in the next sentence. This should be clarified.

Thanks for the comment, we fully rephrase the sentence to make the point more clear.

"[9] like BRENDA [30] or SILVA [31] « those are deposition databases I assume. This could be clarified.

Yes they are ELIXIR deposition databases. We added this fact to the sentence.

-- Infrastructure—
"Figure 2" : The term edal project is a little bit confusing: is it a repository, a software project, all of that ? edal website only could be clearer. Or possibly using software project instead of project alone. This screenshot might need updating.

Yes, you are right this is poorly formulated. Sure, e!DAL is software, but it was of course initially developed in the frame of a project. That's why we named it "project website", but yes this can be confusing. We completely re-formulated the figure caption.

-- Improvements—
"implementation of the e!DAL infrastructure was necessary. " : it is not clear that the following paragraph describe those changes.

Yes, you are right this is ambiguous formulated. We added an additional sentence to make this clear.

-- New Features—
Has Dublin core been cited yet ?
"To add the possibility for assigning an ORCID to every data creator or contributor in the e!DAL infrastructure, the PERSON data type in the metadata schema " : is this the person from an internal metadata scheme or dublinCore/Schemas.org scheme ?

Yes as you assumed this is the metadata attribute from the internal metadata schema, which is inspired by the DublinCore Schema. But in contrast to the DublinCore schema, which does not prescribe any data type for describing the specific attributes, e!DAL bind attributes to dedicated data types. This enables data validity and features the GUI to support users to enter valid metadata elements to describe his datasets. So e.g. the mentioned PERSON data type can be used to set the metadata attributes "Author" or "Contributor". We do not go into detail here, because this was already a part of the first version of e!DAL and our first publication (http://dx.doi.org/10.1186/1471-2105-15-214). We here just focused on the comprehensive update to link the PERSON attribute with the ORCID API. To make this clearer for the reader, we added here again the reference to the first publication and slightly changed the sentence.

"The new e!DAL login module follows the OAuth protocol [40] to authenticate users over the ELIXIR AAI and automatically receive their email address, ": the email address is used as a technical ID of the authenticated user, no ?

Yes you are right, from the conceptual perspective the email address of the user will be used for two aspects. First of all for the communication within the embedded data review process of e!DAL, so that the submitting user gets information about the status of his submission and also receives the final DOI via email. And secondly it is also a kind of "technical" or "internal" ID to authenticate the user within the e!DAL infrastructure.
The second point was from our perspective not so interesting for the reader, but we added an additional sentence into the manuscript to make this clearer.

-- OS specific executables -
Some minor grammar improvement or sentence clarity in this section.
"ava Network Launching Proto- col (JNLP) ": precise that it is the basis of java web start

Thanks for this hint, we corrected this and added a clarification.

--Results—
"Accessible" : the description is true but should be applied to eDale, it is rather general in its current form.

Yes you are right, we reformulated that section to apply this more concrete to e!DAL.

--e!DAL Usage—
"After more than three years of productive usage, the PGP " production?
The last paragraph might be slightly redundant with previous statements.

Yes you are right some points were slightly redundant, we fixed this and changed some sentences in this paragraph.

Close
