# Supplementary material for: The on-premise data sharing infrastructure e!DAL: Foster FAIR data for faster data acquisition
Source: Gigascience. 2020 Oct 22;9(10):giaa107. doi: 10.1093/gigascience/giaa107 (PMC7580168; doi:10.1093/gigascience/giaa107)
Supplement: giaa107_Response_to_Reviewer_Comments_Revision_1 [file giaa107_response_to_reviewer_comments_revision_1.pdf]

Author's Response To Reviewer Comments

Close

Dear Nicole,

Thank you very much for your feedback.
We integrated an additional, new section in the “Result” chapter directly after the “FAIR” part to touch the mentioned points about the semantic metadata and linking to other repositories.

Kind Regards,
Daniel Arend

Close
