# Supplementary material for: The on-premise data sharing infrastructure e!DAL: Foster FAIR data for faster data acquisition
Source: Gigascience. 2020 Oct 22;9(10):giaa107. doi: 10.1093/gigascience/giaa107 (PMC7580168; doi:10.1093/gigascience/giaa107)
Supplement: giaa107_Response_to_Reviewer_Comments_Revision_2 [file giaa107_response_to_reviewer_comments_revision_2.pdf]

Author's Response To Reviewer Comments

Close

Dear Nicole,

we added the requested section/reference.

Close
