# Supplementary material for: The on-premise data sharing infrastructure e!DAL: Foster FAIR data for faster data acquisition
Source: Gigascience. 2020 Oct 22;9(10):giaa107. doi: 10.1093/gigascience/giaa107 (PMC7580168; doi:10.1093/gigascience/giaa107)
Supplement: giaa107_Reviewer_1_Report_Original_Submission — Noah Fahlgren -- 8/11/2020 Reviewed [file giaa107_reviewer_1_report_original_submission.pdf]

Reviewer Report

Title: The on-premise data sharing infrastructure e!DAL: Foster FAIR data for faster data acquisition

Version: Original Submission **Date:** 8/11/2020

Reviewer name: Noah Fahlgren

Reviewer Comments to Author:

Summary

The manuscript "The on-premise data sharing infrastructure e!DAL: Foster FAIR data for faster data acquisition" by Arend and colleagues provides an overview of updates and enhancements to the e!DAL on-premise data-sharing platform. The authors address common issues with scientific data sharing using centralized repositories: 1) data (potentially a large quantity) needs to be transferred from the owner to a repository; 2) data often need to repackaged in various ways to fit the expectations of the repository; 3) dataset metadata needs to potentially be tailored to each repository; 4) costs associated with central repositories (direct or indirect, e.g. training); and 5) lack of central repositories for some data types (e.g. imaging and phenotype data). To address these issues, e!DAL provides the infrastructure to achieve data sharing using FAIR principles using on-premise data infrastructure. e!DAL provides a data publication layer on top of a data repository that interfaces with existing publication and search engine infrastructure to make shared datasets FAIR.

Strengths
----
* The manuscript is well-written and succinctly describes the rationale for and improvements of e!DAL.
* The figures effectively summarize the major features of e!DAL and the results of its usage.

Weaknesses
----
* None

Suggestions for improvement

My understanding is that GPL licenses require an exact copy of the license be distributed with the software: https://www.gnu.org/licenses/gpl-faq.html#WhyMustIInclude. The current license file(s) in e!DAL only states that GPLv3 applies but there does not seem to be a copy of GPLv3 itself.
The manuscript discusses that "By collecting a standardized set of mainly technical metadata e!DAL guarantees a long-term readability and usability of all published datasets." This is important for making datasets findable, but there is an additional layer often required to use data, which is documentation of what the data is, how it was collected, how it is structured, etc. While not in the scope of the current work, could the authors potentially discuss in the outlook section whether they see this type of metadata or documentation being a component of e!DAL? In particular, I am thinking of metadata ontologies like MIAPPE, MIxS, etc.

Level of Interest

Please indicate how interesting you found the manuscript: Choose an item.

Quality of Written English

Please indicate the quality of language in the manuscript: Choose an item.

Declaration of Competing Interests

Please complete a declaration of competing interests, considering the following questions:

- Have you in the past five years received reimbursements, fees, funding, or salary from an organisation that may in any way gain or lose financially from the publication of this manuscript, either now or in the future?
- Do you hold any stocks or shares in an organisation that may in any way gain or lose financially from the publication of this manuscript, either now or in the future?
- Do you hold or are you currently applying for any patents relating to the content of the manuscript?
- Have you received reimbursements, fees, funding, or salary from an organization that holds or has applied for patents relating to the content of the manuscript?
- Do you have any other financial competing interests?
- Do you have any non-financial competing interests in relation to this paper?

If you can answer no to all of the above, write 'I declare that I have no competing interests' below. If your reply is yes to any, please give details below.

I declare that I have no competing interests

I agree to the open peer review policy of the journal. I understand that my name will be included on my report to the authors and, if the manuscript is accepted for publication, my named report including any attachments I upload will be posted on the website along with the authors' responses. I agree for my report to be made available under an Open Access Creative Commons CC-BY license (http://creativecommons.org/licenses/by/4.0/). I understand that any comments which I do not wish to be included in my named report can be included as confidential comments to the editors, which will not be published.

Choose an item.

To further support our reviewers, we have joined with Publons, where you can gain additional credit to further highlight your hard work (see: https://publons.com/journal/530/gigascience). On publication of

this paper, your review will be automatically added to Publons, you can then choose whether or not to claim your Publons credit. I understand this statement.

Yes Choose an item.
