# Supplementary material for: The on-premise data sharing infrastructure e!DAL: Foster FAIR data for faster data acquisition
Source: Gigascience. 2020 Oct 22;9(10):giaa107. doi: 10.1093/gigascience/giaa107 (PMC7580168; doi:10.1093/gigascience/giaa107)
Supplement: giaa107_Reviewer_2_Report_Original_Submission — Guy Cochrane, PhD -- 8/15/2020 Reviewed [file giaa107_reviewer_2_report_original_submission.pdf]

Reviewer Report

Title: The on-premise data sharing infrastructure e!DAL: Foster FAIR data for faster data acquisition

Version: Original Submission **Date:** 8/15/2020

Reviewer name: Guy Cochrane, PhD

Reviewer Comments to Author:

This manuscript provides a clear update on the e!DAL software package for the operation of local data repository infrastructure. Following the "Infrastructure to data (I2D)" model, the tool is available for reuse and is currently operational in two institutions (e.g. operating the "Plant Genomics and Phenomics Data Repository (PGP)" at IPK-Gatersleben) with a further two currently installing it. The manuscript provides details on some appropriate updates and additions that include enhanced data ingress performance, addition of the ORCID identifier system for contributor authentication, the ELIXIR AAI, JSON-LD presentation format, use of Gradle build/deployment infrastructure and a choice of two data upload/submission routes - full application and web application.

e!DAL and its implementations (especially PGP, which could be considered the "reference" implementation), lie within the ELIXIR ecosystem. As the authors note, e!DAL occupies a niche in this ecosystem that is as yet unfilled for many of the data types associated with plant phenomics. However, it also has in scope data of types that can be handled by other elements of the ecosystem, such as the ELIXIR Core Data Resources. These include, for example, genomics databases (see a data set of relevance for this, for example, at https://doi.ipk-gatersleben.de/DOI/1c5dc9c8-0b38-4b2b-93d3-993272532cb1/711ad917-d85b-4e08-b883-8af94ae215b0/2). I recommend that the authors address this issue in the system and the manuscript: how does the system ensure that incoming data sets that include data types appropriate for deposition elsewhere in ELIXIR are appropriately routed and linked from the system?

While promoting FAIR principles, it is not clear from the manuscript how e!DAL supports compliance for data sets with community data standards. I would have expected reference in the text, for example, to ELIXIR-related data standards for plant sciences such as MIAPPE and the Breeding API (https://elixir-europe.org/communities/plant-sciences).

e!DAL takes the I2D model in which distinct repositories operate at institutional level and are connected through the DOI system of identifiers. The authors lay out some but not all of the features of such a model. While they correctly declare that in the event that an e!DAL repository is removed from service, metadata relating to its content will remain in the DataCite system, this is far from optimal as the data themselves will have been lost. In the I2D model, what mechanisms exist, or could be put in place, to protect against such loss?

Level of Interest

Please indicate how interesting you found the manuscript: Choose an item.

Quality of Written English

Please indicate the quality of language in the manuscript: Choose an item.

Declaration of Competing Interests

Please complete a declaration of competing interests, considering the following questions:

- Have you in the past five years received reimbursements, fees, funding, or salary from an organisation that may in any way gain or lose financially from the publication of this manuscript, either now or in the future?
- Do you hold any stocks or shares in an organisation that may in any way gain or lose financially from the publication of this manuscript, either now or in the future?
- Do you hold or are you currently applying for any patents relating to the content of the manuscript?
- Have you received reimbursements, fees, funding, or salary from an organization that holds or has applied for patents relating to the content of the manuscript?
- Do you have any other financial competing interests?
- Do you have any non-financial competing interests in relation to this paper?

If you can answer no to all of the above, write 'I declare that I have no competing interests' below. If your reply is yes to any, please give details below.

I declare that I have no competing interests.

I agree to the open peer review policy of the journal. I understand that my name will be included on my report to the authors and, if the manuscript is accepted for publication, my named report including any attachments I upload will be posted on the website along with the authors' responses. I agree for my report to be made available under an Open Access Creative Commons CC-BY license (http://creativecommons.org/licenses/by/4.0/). I understand that any comments which I do not wish to be included in my named report can be included as confidential comments to the editors, which will not be published.

Choose an item.

To further support our reviewers, we have joined with Publons, where you can gain additional credit to further highlight your hard work (see: https://publons.com/journal/530/gigascience). On publication of this paper, your review will be automatically added to Publons, you can then choose whether or not to claim your Publons credit. I understand this statement.

Yes Choose an item.
