# Supplementary material for: The on-premise data sharing infrastructure e!DAL: Foster FAIR data for faster data acquisition
Source: Gigascience. 2020 Oct 22;9(10):giaa107. doi: 10.1093/gigascience/giaa107 (PMC7580168; doi:10.1093/gigascience/giaa107)
Supplement: giaa107_Reviewer_3_Report_Original_Submission — Cyril Pommier -- 8/20/2020 Reviewed [file giaa107_reviewer_3_report_original_submission.pdf]

Reviewer Report

Title: The on-premise data sharing infrastructure e!DAL: Foster FAIR data for faster data acquisition

Version: Original Submission **Date:** 8/20/2020

Reviewer name: Cyril Pommier

Reviewer Comments to Author:

This paper describe a sustainable data repository solution that provides an intermediate between the most important international data repositories and non-sustainable project databases. It provides an alternative to Research institutions dataverses or dspace with the promise of reduced deployment costs.
This paper therefore discuss a very important question.
The alternate software solutions are fairly presented and the advantages of e!DAL are correctly discussed.
The paper is well written and organized. Some modifications are proposed below.
The software and data repositories are publicly accessible and the software is under GPLv3 open source license. The source code repository is missing a LICENSE file though.
The technical details are clearly introduced and discussed with sufficient information. The reuse of the code is well documented but I haven't tested it.
Therefore the proposition is to request Minor revisions before publishing this quality article.
Remarks:
Some references lack DOI Nature genetics, New Phytologists, …)
DOI minting is not discussed, it could be a plus as getting DOI as a cost for an organization.
A GPLv3 license file should be added in the eDALE code repository.
-- Abstract-- :
Â« the storage of which is not covered by established core databases " : There are established databases, some are Elixir Core for Genomic and others only established for Phenomics.
"Due to its high volume and strong heterogeneity, resulting in missing infrastructures" : this sentence should be clarified.
"ELIXIR AAI Â» : the purpose of this service isn't obvious for non Elixir reader.
"as means tolower Â« : typo
--Introduction--
"general purpose data repositories, e.g. figshare [6], Zenodo [7] and Dryad [8] " : FAIRDOM is another repository that might be worth mentioning.
"(iii) institutional ": research institute might better reflect the authors intention
", e.g. the EBI and NCBI core data resources, Bioinformaticians are charged and trained ": the end of this sentence should be clarified
"or the preparation of ISA-TAB compatible data submission for plant phenotyping data [14, 15]. ": MIAPPE paper: this is the first one, two others have been published since 2015. The latest should be the most accurate.
"Alternatively, project-related or institutional data repositories could be set up. " Can Research

institution repositories and project databases be really placed at the same level ?
"This finally enables the assignment of DOIs with a minimal set of metadata to in-house stored data and its approved FAIR refer- encing by journals or data lookup services. ": it would be worth describing a little be more the metadata (Dublin core minimal dataset or a more extensive list of minimal information about… MIA*)
"Approximately seven million crop accessions Â« : the term accession should be describe a little bit more for new readers (eg PGR accession)
"that do not fit into classical databases due to their volume " : What are precisely those classical databases ? It would be god to refer to the three categories of the first paragraph.
"This experience and the adoption as a service in the European life-sciences Infrastructure for biolog- ical Information ELIXIR [23] " : the words "adoption as a service" would need clarification or rephrasing.
-- Related Work --
"Most of them evolved over many years and they are widely accepted by the research community [24] " the reference 24 is a bit old (2010) if possible a newer one would be a plus.
"Usually also research journals and other pub- lishers request data sharing using these established domain- specific databases. " They require the use of sustainable repositories which can be found using means described in the next sentence. This should be clarified.
"[9] like BRENDA [30] or SILVA [31] Â« those are deposition databases I assume. This could be clarified.
-- Infrastructureâ€"
"Figure 2" : The term edal project is a little bit confusing: is it a repository, a software project, all of that ? edal website only could be clearer. Or possibly usng software project instead of project alone. This screenshot might need updating.
-- Improvementsâ€"
"implementation of the e!DAL infrastruc- ture was necessary. " : it is not clear that the following paragraph describe those changes.
-- New Featuresâ€"
Has Dublin core been cited yet ?
"To add the possibility for assigning an ORCID to every data creator or contributor in the e!DAL infrastructure, the PERSON data type in the metadata schema " : is this the person from an internal metadata scheme or dublinCore/Schemas.org scheme ?
"The new e!DAL login module follows the OAuth protocol [40] to authenticate users over the ELIXIR AAI and automat- ically receive their email address, ": the email address is used as a technical ID of the authenticated user, no ?
-- OS specific executables -
Some minor grammar improvement or sentence clarity in this section.
"ava Network Launching Proto- col (JNLP) ": precise that it is the basis of java web start
--Resultsâ€"
"Accessible" : the description is true but should be applied to eDale, it is rather general in its current form.
--e!DAL Usageâ€"
"After more than three years of productive usage, the PGP " production?

The last paragraph might be slightly redundant with previous statements.

Level of Interest

Please indicate how interesting you found the manuscript: Choose an item.

Quality of Written English

Please indicate the quality of language in the manuscript: Choose an item.

Declaration of Competing Interests

Please complete a declaration of competing interests, considering the following questions:

- Have you in the past five years received reimbursements, fees, funding, or salary from an organisation that may in any way gain or lose financially from the publication of this manuscript, either now or in the future?
- Do you hold any stocks or shares in an organisation that may in any way gain or lose financially from the publication of this manuscript, either now or in the future?
- Do you hold or are you currently applying for any patents relating to the content of the manuscript?
- Have you received reimbursements, fees, funding, or salary from an organization that holds or has applied for patents relating to the content of the manuscript?
- Do you have any other financial competing interests?
- Do you have any non-financial competing interests in relation to this paper?

If you can answer no to all of the above, write 'I declare that I have no competing interests' below. If your reply is yes to any, please give details below.

I declare that I have no competing interests

I agree to the open peer review policy of the journal. I understand that my name will be included on my report to the authors and, if the manuscript is accepted for publication, my named report including any attachments I upload will be posted on the website along with the authors' responses. I agree for my report to be made available under an Open Access Creative Commons CC-BY license (http://creativecommons.org/licenses/by/4.0/). I understand that any comments which I do not wish to be included in my named report can be included as confidential comments to the editors, which will not be published.

Choose an item.

To further support our reviewers, we have joined with Publons, where you can gain additional credit to further highlight your hard work (see: https://publons.com/journal/530/gigascience). On publication of this paper, your review will be automatically added to Publons, you can then choose whether or not to claim your Publons credit. I understand this statement.

Yes Choose an item.
